# Angiotensin receptor blockers retard the progression and fibrosis via inhibiting the viability of ^AGTR1+^CAFs in intrahepatic cholangiocarcinoma

**DOI:** 10.1002/ctm2.1213

**Published:** 2023-02-28

**Authors:** Jian‐Hui Li, Xiao Wu, Xuhao Ni, Ya‐Xiong Li, Long Xu, Xiao‐Yi Hao, Wei Zhao, Xiao‐Xu Zhu, Xiao‐Yu Yin

**Affiliations:** ^1^ Department of Pancreato‐Biliary Surgery The First Affiliated Hospital Sun Yat‐sen University Guangzhou Guangdong China; ^2^ Key Laboratory of Stem Cells and Tissue Engineering Sun Yat‐sen University Ministry of Education Guangzhou Guangdong China; ^3^ Lau Luen Hung Private Medical Center Unit 3 (Surgery) The First Affiliated Hospital Sun Yat‐sen University Guangzhou Guangdong China; ^4^ Department of Physiology, Zhongshan School of Medicine Sun Yat‐Sen University Guangzhou Guangdong China

**Keywords:** AGTR1, angiotensin receptor blockers, cancer‐associated fibroblasts, intrahepatic cholangiocarcinoma, MFAP5

## Abstract

**Background:**

Intrahepatic cholangiocarcinoma (iCCA) is a highly lethal malignancy characterized by massive fibrosis and has ineffective adjuvant therapies. Here, we demonstrate the potential of angiotensin receptor blockers (ARBs) in targeting iCCA.

**Methods:**

Masson's trichrome staining was used to assess the effect of ARBs in iCCA specimens, CCK8 and gel contraction assays in vitro and in xenograft models in vivo. RNA‐seq and ATAC‐seq were used for mechanistic investigations.

**Results:**

Patients with iCCA who were administered ARBs had a better prognosis and a lower proportion of tumour stroma, indicating alleviated fibrosis. The presence of AGTR1, the ARBs receptor, is associated with a poor prognosis of iCCA and is highly expressed in tumour tissues and cancer‐associated fibroblasts (CAFs). The ARBs strongly attenuated the viability of ^AGTR1+^CAFs in vitro and retarded tumour progression and fibrosis in xenograft models of co‐cultured CAFs and iCCA cells. Still, they did not have a significant effect on ^AGTR1−^CAFs. Moreover, ARBs decreased the secretion of ^AGTR1+^CAF‐derived MFAP5 via the Hippo pathway, weakened the interaction between CAFs and iCCA cells, and impaired the aggressiveness of iCCA cells by attenuating the activation of the Notch1 pathway in iCCA cells.

**Conclusions:**

ARBs exhibit anti‐fibrotic function by inhibiting the viability of ^AGTR1+^CAFs. These findings support using ARBs as a novel therapeutic option for targeting iCCA.

## 1 BACKGROUND

Intrahepatic cholangiocarcinoma (iCCA) is a subgroup of cholangiocarcinoma (CCA), which originate from the epithelial cells of the proximal to second‐order bile ducts.^1^ The surging incidence of iCCA over the last 10–20 years has become a significant challenge for clinicians.^2^ Radical surgery is still the best option for iCCA, but only approximately 30% of patients with iCCA are appropriate contenders at the time of diagnosis. Patients who underwent radical surgery had a recurrence rate of 40%–80%, and fewer than one‐third of the patients survived beyond 5 years.^3^ Although studies have provided many molecular targets and systemic therapies for iCCA treatment in the past few years, only limited treatment effects have been achieved. Therefore, it is imperative to develop effective approaches to improving the prognosis of patients with iCCA.

The subgroup, iCCA, is characterized by extensive fibrosis in tumour tissues. The stroma comprises a large proportion of cancer‐associated fibroblasts (CAFs) and their secretions, which closely surround tumour cells.^4^ The CAFs produce extracellular matrix (ECM) components and communicate with endothelial, immune and cancer cells via interactive signaling pathways, thus facilitating tumour progression by angiogenesis, immunosuppression and proliferation.^5^ Huge number of fibroblasts infiltrating the tumour microenvironment (TME) is reportedly a positive predictor for metastatic potential and poor prognosis.^6^ Accumulating data have indicated that an imbalance in the renin‐angiotensin system (RAS) facilitates malignancy and correlates with poor patient outcomes for various cancers.^7^ The RAS plays a crucial role in controlling blood pressure, and angiotensin II (Ang II) is one of the most vital components of RAS. Tissues from iCCA had high Ang II concentrations. This locally formed Ang II was associated with activated hepatic stellate cells (HSCs), which were reported as one of the original CAF cells. The Ang II/AGTR1(Ang II receptor) axis is suggested to have autocrine and paracrine synergistic effects on tumour progression and cancer fibrogenesis.^8^ Angiotensin receptor blockers (ARBs), which selectively block the activation of AGTR1, are reported to have an anti‐fibrotic function and have been considered a promising therapeutic approach for lung fibrosis, intrauterine adhesions and renal fibrosis.^9^, ^10^, ^11^ The ARBs are also reportedly instrumental in inhibiting liver fibrogenesis in animal models.^12^ However, the effects and mechanisms of action of ARBs in iCCA fibrosis remain unreported.

In the present study, we revealed that ARBs administration was beneficial to the prognosis of patients with iCCA. ARBs attenuated the viability of ^AGTR1+^CAFs and exhibited an anti‐fibrotic effect on the iCCA cells. Further results demonstrated that ARBs inhibited the secretion of ^AGTR1+^CAF‐derived MFAP5 through the Hippo pathway, thereby attenuating activation of the Notch1 pathway and impairing the aggressiveness of the iCCA cells. Thus, our findings indicate that ARBs may be a potent treatment option for iCCA.

## 2 METHODS

### 2.1 Patient specimens

Ninety‐one iCCA patients with hypertension who underwent hepatectomies between January 2014 and December 2021 were recruited for the prognosis analysis. The inclusion criteria were as follows: (i) patients with localized iCCA (pT1a‐3N0M0), (ii) those who underwent radical hepatectomy, (iii) those who survived longer than 3 months after hepatectomy, and (iv) those with comprehensive clinicopathological data and follow‐up information. The flow diagram (Figure S1A) shows that 141 patients were concurrently diagnosed with iCCA and hypertension and underwent surgery in our centre between January 2014 and December 2021. Twenty‐five patients were lost to follow‐up, and 25 patients with TNM stage greater than IIIa were excluded from the study. Finally, 91 patients were enrolled in the study cohort, including 71 receiving anti‐hypertensive drugs. The clinicopathological characteristics of the 91 patients with iCCA are shown in Table S1, and the details of the 71 patients who were administered antihypertensive drugs are shown in Table S2. Paraffin‐embedded specimens from these 91 patients were used for subsequent HE, Masson's and IHC assays. The detailed operational procedure for measuring tumour stroma using ImageJ software has been described previously.^13^

### 2.2 Primary CAFs isolation

CAFs #1–6 were isolated from iCCA tumour tissues. The normal fibroblast #1 (NF‐1) was obtained from the bile duct without cancer cells. This isolation assay was performed as previously described.^13^ Briefly, fresh specimens collected from the operation theatre were preserved in Hank's salt solution (#SH30588.01, Hyclone). The tissue was then separated into small pieces and incubated with DNAse I (10 μg/mL; #D5025‐15KU, Sigma‐Aldrich), collagenase I (200 U/mL; #LS004197, Worthington Biochem) and collagenase P (0.5 mg/mL; #11213865001, Roche) in Hank's salt solution for 30 min at 37°C and 200 rpm. A 100‐mm cell filter was used to filter the solution. Subsequently, the solution was washed several times in a 70‐mm cell filter using FAST buffer (0.2% BSA and 2 mM EDTA in PBS). To isolate the fibroblast populations, the cell suspensions were incubated with anti‐fibroblast MicroBeads (130‐050‐601, Miltenyi Biotec) in FAST buffer at 4°C for 15 min and then applied to LS columns (130‐042‐401) in a QuadroMACS Separator (130‐090‐976). A total of 5–10 passages of primary fibroblasts were used in the experiments.

### 2.3 Cell culture and transduction

The human iCCA cell lines, RBE and HuCC‐T1, were purchased from Cellcook Cell Biotechnology (Guangzhou, China). The cells were cultured in RPMI‐1640 (Cellcook, #L210KJ), supplemented with 10% fetal bovine serum (FBS; Gibco, #A3160802). Losartan (#HY‐17512), valsartan (#HY‐18204), angiotensin II (HY‐13948) and XMU‐MP‐1 (HY‐100526) were purchased from MedChemExpress (Shanghai, China). Cell culture inserts of 0.4 μm PC pore size in six‐well plates (Thermo Scientific 140640) were implemented in the tumour cell/CAF co‐culture system. The iCCA cells (5 × 10^4^) were seeded in six‐well plates, and CAFs (1 × 10^5^) were seeded into the inserts. For MFAP5 knockdown assays, CAFs were transfected with a lentivirus‐encoding shRNA. The shRNA sequences used for silencing the MFAP5 and control (TRC) are provided in Table S3.

### 2.4 Xenograft assay

The BALB/c nude mice (4–6 weeks old) were purchased from the laboratory animal centre of Sun Yat‐sen University. Mice were injected subcutaneously with 2 × 10^6^ HuCC‐T1 cells mixed with 4 × 10^6^ CAFs. Five biological replicates were used for each treatment group. The tumours were measured every 3 days. The formula for tumour volume calculation was 0.5 × length × width × height. Tumour‐bearing mice were sacrificed when the maximum volume reached 1500 mm^3^, and the tumours were harvested for further study.

### 2.5 Quantitative real‐time PCR assays

TRIzol reagent (Life Technologies, USA) was used to extract the total RNA. Reverse transcription was performed using the HiScript II Q RT SuperMix for qPCR (#R223‐01, Vazyme). The qRT‐PCR was performed on a Life Quant Studio 6 Flex Real‐time PCR system using the ChamQ Universal SYBR qPCR Master Mix (#Q711‐02, Vazyme). All primers used in the experiments are listed in Table S3.

### 2.6 Western blot analysis

The cell lysate was harvested using the Pierce lysis buffer supplemented with a phosphatase inhibitor cocktail. Cytosolic and nuclear protein lysates were extracted using a Bestbio kit (#BB‐3104‐2). Immunoblotting was performed with the following primary antibodies: AGTR1 (#25343‐1‐AP, Proteintech), MFAP5 (#15727‐1‐AP, Proteintech), Notch activated target antibody sample kit (#68309, cst), LATS1 (#17049‐1‐AP, Proteintech), Phospho‐LATS1 (#AF7170, affinity), YAP1 (#205270, Abcam), Phospho‐YAP1 (#76252, Abcam), H3 (#1797, Abcam), GAPDH (#10494‐1‐AP, Proteintech) and β‐actin (#4970, cst) in a universal antibody diluent (#WB500D, NCM) overnight. After incubation with an anti‐rabbit IgG‐HRP‐conjugated secondary antibody (#7071, cst) for 1 h, the signals were visualized using the Immobilon Western Blotting substrate (Millipore, USA).

### 2.7 Enzyme‐linked immunosorbent assay

An enzyme‐linked immunosorbent assay (ELISA) Kit (SEF590Hu Cloud‐Clone Corp) was used to detect the protein level of MFAP5 in the cell supernatant.

### 2.8 Flow cytometry

The CAFs were prepared, fixed and incubated with AGTR1 (#FAB10244G, R&D Systems) for fluorescence‐activated cell sorting analysis. Flow cytometry was performed using a BD FACS Aria III (BD Biosciences, CA, USA). The analysis was performed using FlowJo v10.8.1.

### 2.9 RNA‐seq and ATAC‐seq assays

An RNA‐seq assay of CAFs^AGTR1(+)^ was performed. The gene count data were normalized using the relative log expression method, which accounts for RNA‐seq library size differences. A gene was defined as DEGs if the Benjamini–Hochberg adjusted *p*‐value was less than .05 and fold changes were greater than or equal to 2. A gene ontology (GO) functional enrichment analysis was conducted using the R packages GOSeq (version 1.10.0) and topGO (version 2.10.0).^14^ The DEGs were significantly enriched (in GO terms) when their Bonferroni‐corrected *p*‐values were less than or equal to .05. The gene set enrichment analysis (GSEA) presented in this study were performed using Java GSEA.^15^ The alignment background was referred to as the Molecular Signatures Database v7.5. As described previously, an assay for transposase‐accessible chromatin with high‐throughput sequencing (ATAC‐seq) was performed,^16^ and is showcased in Supporting Methods.

### 2.10 Viability assays of CAFs and tumour cells

The CAFs were seeded into 96‐well plates at a density of 3 × 10^3^ per well. The proliferation ability of the CAFs was detected at 0, 24, 48 and 72 h after their respective interventions with the cell counting kit 8 (CCK8) (Dojindo, Kumamoto, Japan). Approximately 5 × 10^6^ CAFs were seeded into 10‐cm cell culture dishes for the tumour cell assays and cultured with dimethyl sulfoxide (DMSO) complete medium. After 48 h, the CAFs cell culture medium was removed, filtered by a 0.2 μm filter and mixed with the original culture medium, at a 1:1 ratio (conditioned medium). Tumour cell viability was detected using the same method as the CAFs. Absorbance values at 450 nm were recorded as a representation of cell viability.

### 2.11 Plate clone formation assay

Four hundred tumour cells were seeded per well into a six‐well plate and cultured for 20 days. The conditioned medium was changed every 4 days. The cells were then fixed in 4% formaldehyde for 20 min and stained with 0.5% crystal violet for 30 min. Plates were washed twice using PBS, pictures were taken, and colony numbers were calculated using ImageJ.

### 2.12 Half‐maximal inhibitory concentration

The CAFs were seeded into a 96‐well plate at a density of 5.0 × 10^3^ cells per well. After 24 h of incubation, the cell medium was replaced with 100 μL of fresh medium containing different concentrations of losartan or valsartan. Cells treated with DMSO were used as controls. The cells were cultured for 48 h and then incubated for 2 h at 37°C with CCK8 solution, followed by quantification at 450 nm using a microplate reader. The half‐maximal inhibitory concentration (IC_50_) was analyzed using GraphPad Prism 9.4 software (La Jolla, CA, USA).

### 2.13 Gel contraction assays

To quantify active gel contraction, the CAFs cells (2.5 × 10^5^ cells) were embedded in 500 μL of collagen type I (#354249, Corning)/Matrigel (#354230, Corning). Final concentrations of collagen (2 mg/mL) and Matrigel (1 mg/mL) were obtained from a 24‐well plate. The gel‐polymerized (30 min) cells were washed once in 10% FBS DMEM for 1 h and then replaced with fresh media containing the different treatments. The gel contraction was monitored at 8, 24 and 48 h by taking photographs under ChemiDOC XRS. The surface area of the gel was measured using ImageJ software, and the percentage of contraction was calculated using the following formula: 100 × (well area − gel area)/well area.

### 2.14 Statistical analysis

Statistical analyses were performed using GraphPad Prism 9.4 (La Jolla, CA, USA) and the SPSS software (version 26.0; IBM, Chicago, IL, USA). Comparisons were performed using either a two‐tailed *t*‐test or one‐way ANOVA. Quantified values are reported as the mean ± SD. *p* < .05 was considered as statistically significant (*p < .05, **p < .01, ***p < .001, ****p < .0001).

## 3 RESULTS

### 3.1 Benefits of ARB administration in patients with iCCA

**FIGURE 1:**
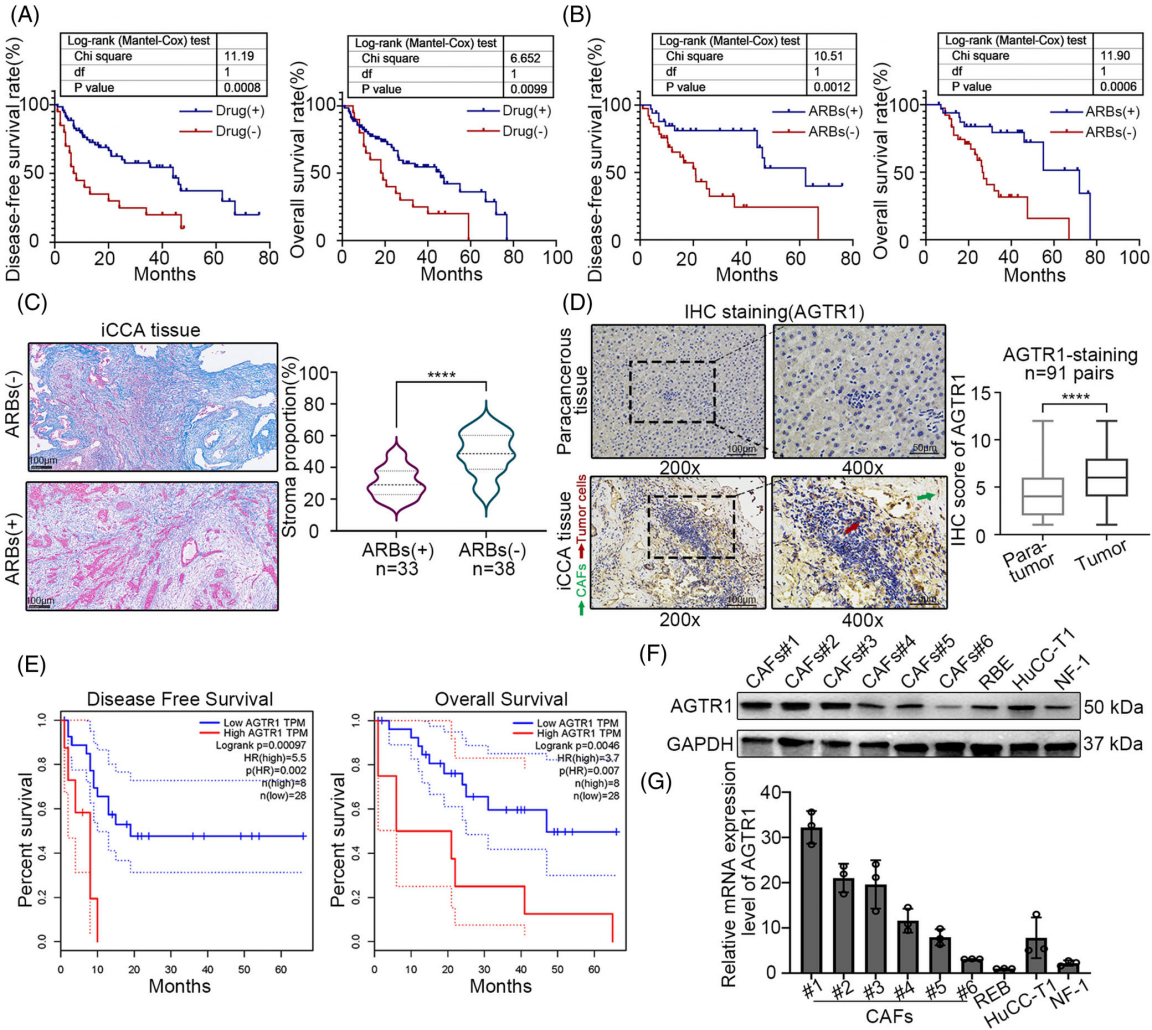
Benefits of angiotensin receptor blocker (ARB) administration to patients with intrahepatic cholangiocarcinoma (iCCA). (A) Survival analysis based on antihypertensive drug usage in iCCA patients (*n* = 71, drug(+); *n* = 20, drug(−)). (B) Survival analysis based on ARBs usage in iCCA patients (*n* = 38, ARBs(+); *n* = 33, ARBs(−)). (C) Masson's trichrome staining of iCCA tissues with ARBs(−) or ARBs(+) administration (scale bar = 100 μm). (D) IHC results showed the protein level of AGTR1 in iCCA and paracancerous tissues (*n* = 91). (E) Survival analysis based on AGTR1 expression in cholangiocarcinoma tissues from TCGA database (*n* = 36, cut‐off value: upper 20%, AGTR1 = 12.8014). (F) Western blot results showed the protein level of AGTR1. (G) qRT‐PCR results showed the mRNA level of AGTR1. Data are presented as mean ± SD and compared by *t*‐test. *p < .05, **p < .01, ***p < .001, ****p < .0001.

To validate whether ARBs were associated with iCCA prognosis, we analyzed clinical data from 91 patients with combined, localized iCCA (pT1a‐3N0M0) and hypertension. The patient's clinical characteristics and anti‐hypertensive drug use are shown in Tables S1 and S2, respectively. First, patients were categorized into two groups according to whether they took anti‐hypertensive drugs; drug(+), *n* = 71 and drug(−), *n* = 20. The Kaplan–Meier curves showed that disease‐free survival (DFS) and overall survival (OS) rates were significantly higher for the drug(+) group than for the drug(−) group (*p* < .05, Figure 1A). Subsequently, subgroup analysis was performed within the drug(+) group based on the categories of anti‐hypertensive drugs. Between the RAS inhibitor(+) (*n* = 37) and RAS inhibitor(−) (*n* = 34) groups, the results showed that the DFS (*p* = .0106) and OS (*p* = .0045) rates were higher for the RAS inhibitor (+) group (Figure S1B). Both DFS and OS rates were significantly higher for the ARBs(+) (*n* = 33) and ARBs(−) (*n* = 38) groups, but higher in the ARBs(+) group (*p* < .05, Figure 1B). Univariate and multivariate regression analyses showed that the administration of anti‐hypertensive drugs and ARBs were independent prognostic factors for DFS and OS (Table 1). These results indicated that the administration of ARBs benefited the prognosis of patients with iCCA. Next, we explored how ARBs retard iCCA progression. The ARBs reportedly reduce ECM content and associated fibrosis in ovarian cancer.^17^ Tumour fibrosis and angiotensin‐driven fibrogenic signaling are inversely correlated with survival. Inspired by this, we examined the effect of ARBs on the proportion of fibrosis in iCCA tissues. Masson's trichrome staining showed that the proportion of stroma was much higher in the ARBs(−) group than in the ARBs(+) group (Figure 1C). These results suggested that ARBs retarded iCCA progression through their antifibrotic function.

**TABLE 1:** Risk factor for DFS and OS of 91 iCCA patients following radical operation

Variable (patient number)	Disease‐free survival			Overall survival		
	HR	95% CI	*p*‐Value	HR	95% CI	*p*‐Value
Univariate						
Age (>64, *n* = 43/≤64, *n* = 48)	1.15	0.65–2.03	.618	1.01	0.63–1.94	.732
Gender (female, *n* = 36/male, *n* = 55)	0.78	0.43–1.40	.376	0.71	0.39–1.29	.224
Hypertension						
Administrated drugs (yes, *n* = 71/no, *n* = 20)	0.39	0.18–0.81	.0008	0.39	0.19–0.82	.0009
Administrated ARBs drugs (yes, *n* = 33/no, *n* = 38)	0.34	0.16–0.68	.0012	0.32	0.16–0.65	.0006
AGTR1^a^ protein level (≤6, *n* = 51, >6, *n* = 40)	0.41	0.23–0.73	.0018	0.39	0.22–0.70	.0008
Tumour differentiation						
Well vs. moderately (*n*, 15 vs. 51)	0.57	0.25–1.28	.105	0.58	0.26–1.29	.108
Well vs. poorly (*n*, 15 vs. 7)	0.68	0.26–1.77	.419	0.72	0.28–1.88	.497
Moderately vs. poorly (*n*, 51 vs. 7)	0.78	0.30–2.03	.565	0.82	0.32–2.11	.653
8th AJCC TNM						
I vs. II (*n*, 52 vs. 19)	1.05	0.49–2.26	.903	1.02	0.47–2.21	.969
II vs. IIIa (*n*, 19 vs. 20)	0.71	0.29–1.74	.449	0.67	0.27–1.66	.379
I vs. IIIa (*n*, 52 vs. 20)	0.51	0.24–1.08	.003	0.53	0.25–1.10	.037
Multivariate						
Administrated drugs (yes, *n* = 71/no, *n* = 20)	0.38	0.21–0.70	.001	0.40	0.21–0.72	.002
Administrated ARBs drugs (yes, *n* = 33/no, *n* = 38)	0.25	0.12–0.52	.000	0.21	0.19–0.45	.000
AGTR1 protein level (≤6, *n* = 51, >6, *n* = 50)	0.47	0.49–1.06	.002	0.44	0.47–1.01	.001
8th AJCC TNM (I/IIIa) (*n*, 52 vs. 20)			.315			.277

Abbreviations: ARBs, angiotensin receptor blockers; CI, confidence interval; DFS, disease‐free survival; HR, hazard ratio; iCCA, intrahepatic cholangiocarcinoma; OS, overall survival.

^a^ Immunohistochemical (IHC) score, split at median.

### 3.2 ARBs attenuated the viability of ^AGTR1+^CAFs

**FIGURE 2:**
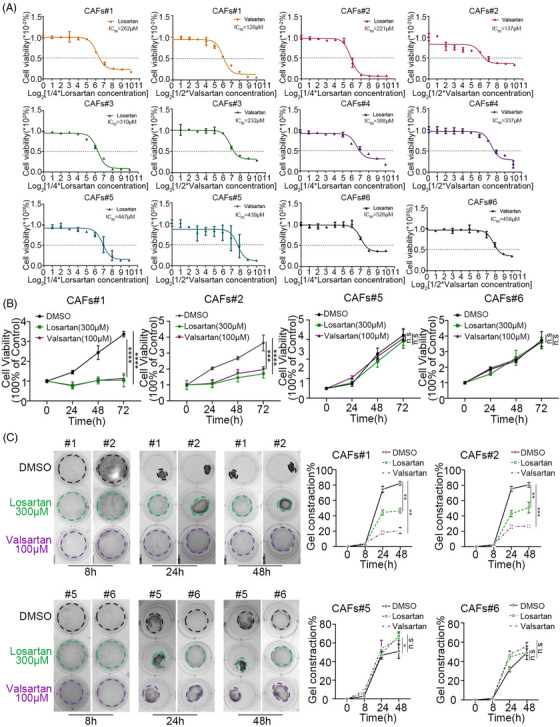
Cancer‐associated fibroblasts (CAFs) responded differently to angiotensin receptor blockers (ARBs). (A) Cell viability results of CAFs 48 h after administering gradient concentrations of ARBs (losartan and valsartan). Fitting curves showed the IC_50_ values of ARBs in CAFs #1–6, respectively. (B) Cell viability results showed a different proliferation rate after administrating losartan (300 μM) or valsartan (100 μM) in CAFs #1, #2, #5 and #6. (C) Gel contraction results of CAFs with losartan or valsartan administration at different time points. Left panel: contracted gel within black, green and purple lines represented DMSO, losartan, and valsartan, respectively. Right panel: quantification of gel contraction rate under losartan and valsartan. Data are presented as mean ± SD (*n* = 3) and compared by *t*‐test. *p < .05, **p < .01, ***p < .001, ****p < .0001, n.s. = no statistical differences.

Pharmacologically, ARBs can selectively block the action of AGTR1. The IHC analysis showed that the intensity of positive staining for AGTR1 was higher in iCCA tissues than in paracancerous tissues (Figure 1D). Kaplan–Meier curves from the TCGA database showed that the DFS and OS of patients with iCCA with high expression levels of AGTR1 were significantly lower than those with low expression levels (*p* < .05, Figure 1E). With a cut‐off value of 12.8014 (FPKM) (Figure S1C,D), which separated the entire group into low (28) and high (8) subgroups, both OS and DFS were significantly different. In addition, 91 patients with iCCA at our centre were categorized into two groups according to their median AGTR1 level (IHC score): high‐level group (AGTR1 > 6, *n* = 40) and low‐level group (AGTR1 ≤ 6, *n* = 51). These two groups had significant differences in OS and DFS (Figure S1E,F). The poor prognosis of solid tumours is inseparable from the high proportion of stroma. Masson's trichrome staining was performed on iCCA tissues, and we observed that the proportion of stroma was much higher in iCCA tissues than in paracancerous tissues (Figure S1G). Combining these results, we deduced that AGTR1, the target of ARBs, was associated with the fibrosis of iCCA tissues. CAFs are identified as one of the major cell‐producing components of ECM in various solid tumours. We hypothesized that AGTR1 might be highly expressed in CAFs. To verify this hypothesis, we isolated CAFs #1–6 from iCCA tumour tissues, and the microscopic morphologies are shown in Figure S2A. The purity of the fibroblasts was validated by immunofluorescence staining, which showed that the fibroblasts were positive for α‐SMA (>95%) (Figure 2B,C). The results of Western blotting and qRT‐PCR confirmed that the expression level of AGTR1 was generally higher in CAFs than in tumour cells (Figure 1F,G and Figures S1H,I and S12A). And the AGTR1 expression was not significantly biased between passages 5 and 10 for CAFs (Figure S14A–F). After analyzing the public scRNA‐seq data (GSE138709), we found that the expression of AGTR1 was generally greater in fibroblasts than in epithelial cells (Figure S3A–F). The results indicated that ARBs might retard iCCA progression by blocking the AGTR1 in CAFs.

**FIGURE 3:**
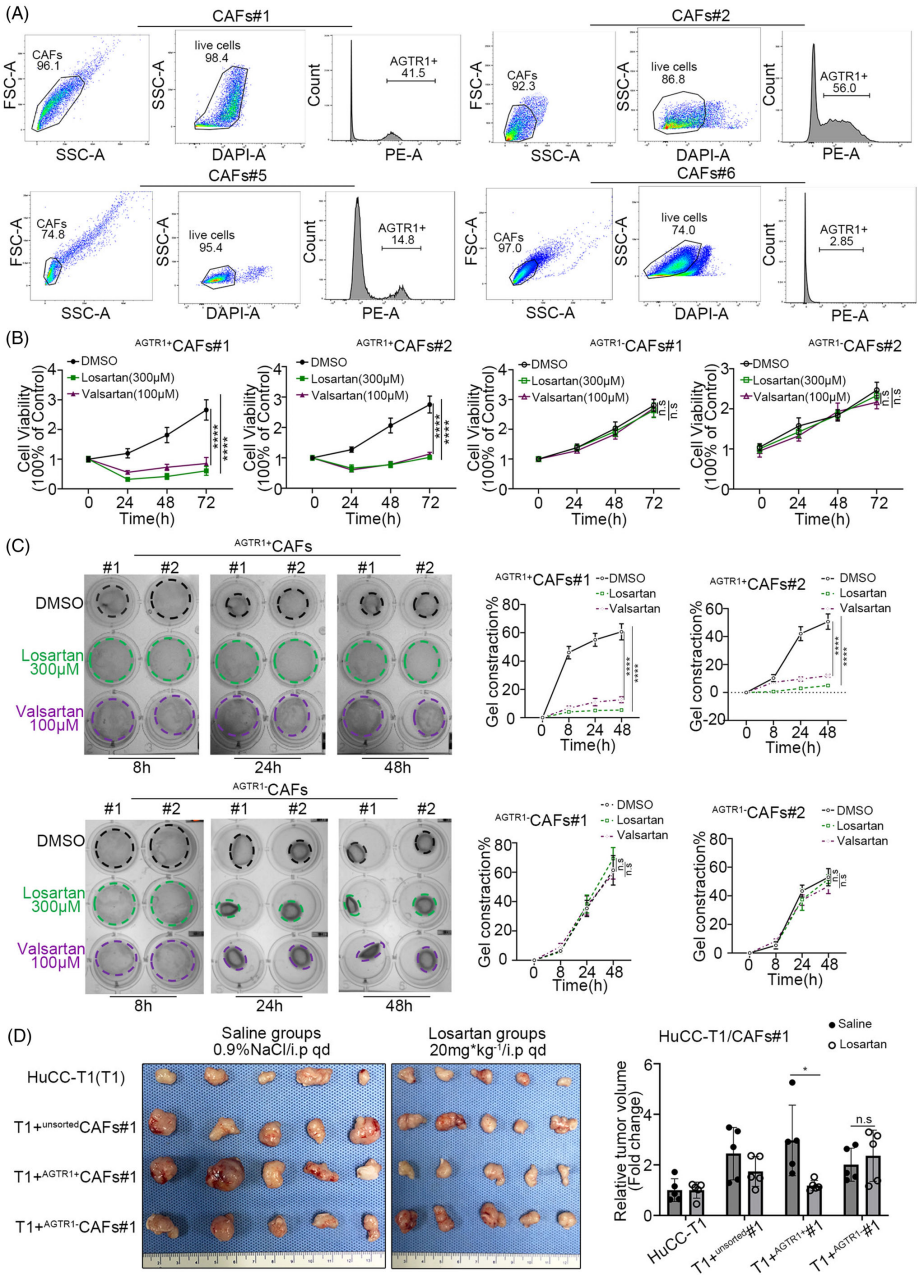
Angiotensin receptor blockers (ARBs) attenuated the viability and contractility of ^AGTR1+^CAFs (cancer‐associated fibroblasts). (A) Flow cytometry results of CAFs treated with AGTR1 antibody (^AGTR1+^CAFs accounted for 41.5%, 56.0%, 14.8% and 2.85% in CAFs #1, #2, #5 and #6, respectively). (B) Cell viability results showed a different proliferation rate after administrating losartan (300 μM) or valsartan (100 μM) in ^AGTR1+/−^CAFs #1 and #2. (C) Gel contraction results of ^AGTR1+/−^CAFs #1 and #2 with losartan or valsartan administration at different time points. Left panel: contracted gel within black, green and purple lines represented DMSO, losartan and valsartan, respectively. Right panel: quantification of gel contraction rate under losartan and valsartan. (D) The relative tumour volume of xenograft tumours (*n* = 5) with saline or losartan administration in the respective groups. Data are presented as mean ± SD (*n* = 3) and compared by *t*‐test. *p < .05, ****p < .0001, n.s. = no statistical differences.

To verify the effect of ARBs on CAFs, we tested the IC_50_ values of ARBs (losartan and valsartan) in iCCA‐CAFs (Figure 2A). The different values of IC_50_ represent different sensitivities of CAFs to ARBs. Furthermore, the CAFs were divided into ARB‐sensitive (low IC_50_ value, CAFs #1–3) and ARB‐insensitive (high IC_50_ value, CAFs #4–6) groups. The results of the CCK8 assay suggested that after ARBs administration, the cell viabilities of CAFs #1–3 were significantly lower than that of CAFs #4–6 (Figure 2B and Figure S3G,I). The gel contraction assay showed that ARBs significantly attenuated the contractility of CAFs #1–3 but did not have much effect on CAFs #4–6 (Figure 2C and Figure S3H,J). The protein levels of AGTR1 were higher in CAFs #1–3 than in CAFs #4–6 (Figure 1F and Figure S13A), and CAFs were characterized as highly heterogeneous populations of cells. Thus, we speculated that the sensitivity of CAFs to ARBs was positively correlated with the proportion of ^AGTR1+^CAFs. Flow cytometric cell sorting (FACS) was used to verify this hypothesis by testing the proportion of ^AGTR1+^CAFs and ^AGTR1−^CAFs in CAFs #1–6. The FACS results confirmed that the proportion of ^AGTR1+^CAFs in CAFs #1–3 (CAFs #1: 41.5%; CAFs #2: 56.0%; CAFs #3: 36.3%) was significantly higher than that in CAFs #4–6 (CAFs #4: 17.0%; CAFs #5: 14.8%; CAFs #6: 2.85%) (Figure 3A and Figure S4A). These findings correlated with Western blotting results (Figure S4B). The FACS results showed that CAFs could be separated into two groups by AGTR1, a membrane protein. This was confirmed by analyzing the public scRNA‐seq data (GSE142784) (Figure S9E–G). We then sorted CAFs #1 and #2 into ^AGTR1+^CAFs and ^AGTR1−^CAFs and tested the effect of ARBs on them. Results of the CCK8 and gel contraction assays showed that ARBs significantly attenuated the cell viability and contractility ability of ^AGTR1+^CAFs compared with ^AGTR1−^CAFs (Figure 3B,C and Figure S5A–D). We then performed a xenograft assay with a mixed injection of iCCA tumour cells (HuCC‐T1 [T1], RBE) and CAFs (^unsorted^CAFs #1, ^AGTR1+^CAFs #1, ^AGTR1−^CAFs #1) in BALB/c nude mice. The tumour volume of T1+^AGTR1+^CAFs/RBE+^AGTR1+^CAFs showed the most significant decrease after treatment with ARBs (Figure 3D and Figure S5E–G). This decrease also applied to the stromal proportion (Figure S6A), indicating that ^AGTR1+^CAFs directly contributed to fibrosis. Moreover, between groups with unsorted CAFs, the proportion of stroma in the saline group was retained in the tumour microenvironment, but significantly decreased after treatment with ARBs (Figure S6C), which was correlated with the proportion of ^AGTR1+^CAFs (Figure S6B). In summary, we demonstrated that ARBs attenuate fibrosis in iCCA by inhibiting the viability and contractility of ^AGTR1+^CAFs.

### 3.3 ARBs might be involved in the regulation of MFAP5 secretion by ^AGTR1+^CAFs

**FIGURE 4:**
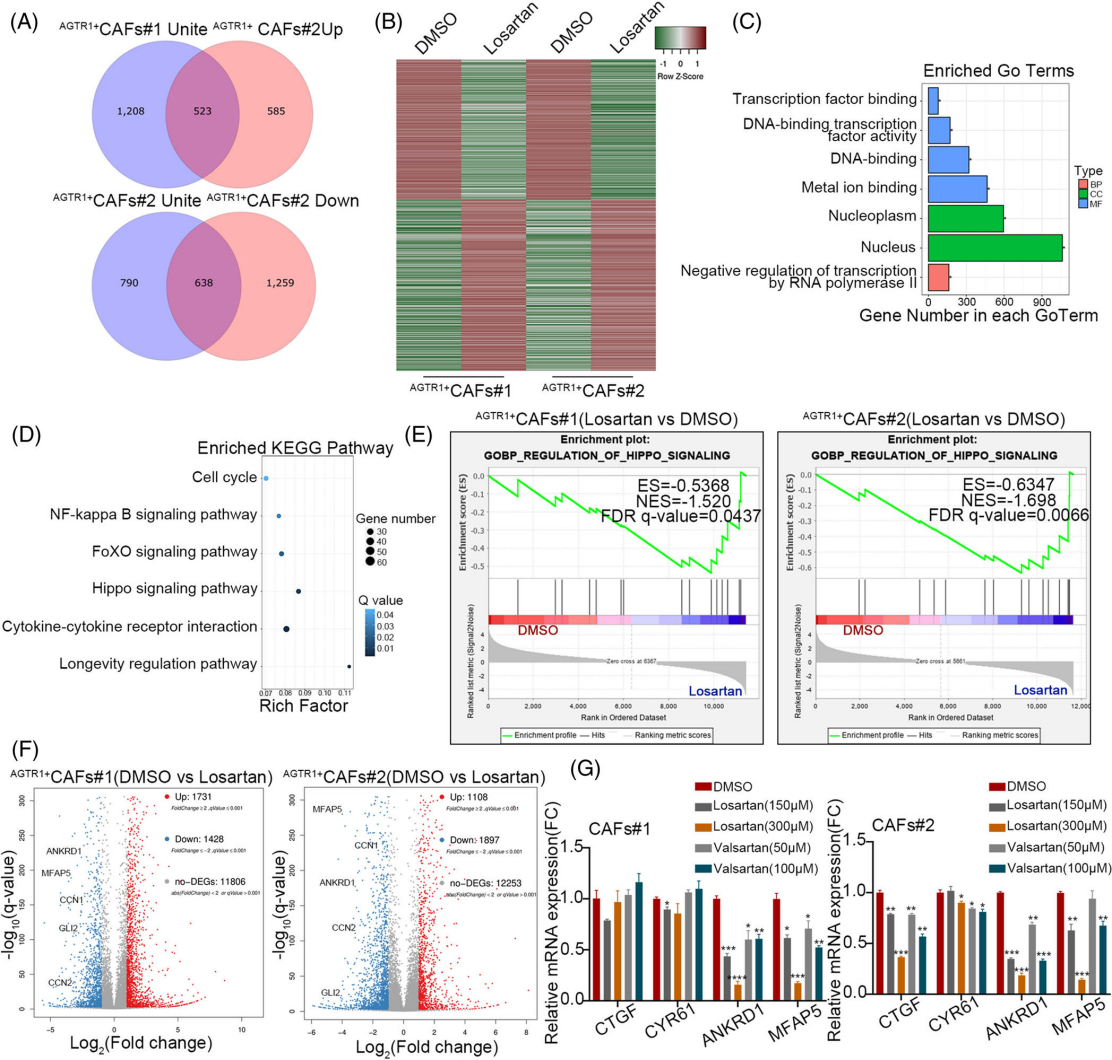
Hippo pathway inhibition after administration of angiotensin receptor blockers (ARBs) in ^AGTR1+^CAFs (cancer‐associated fibroblasts). (A) Venn diagram representing 638 downregulated and 523 upregulated genes differently expressed after administrating losartan in ^AGTR1+^CAFs. (B) Heatmap of 1161 differentially expressed genes in DMSO and losartan (fold change >2 or < −2, *p* < .01). (C and D) Gene ontology (GO) and Kyoto Encyclopedia of Genes and Genomes (KEGG) annotation using genes where the differential expressions were significant. (E) Gene set enrichment analysis (GSEA) results show downregulation of the Hippo pathway in ^AGTR1+^CAFs administered with losartan compared with DMSO. (F) Volcano plot shows the distribution of differentially upregulated (red) and downregulated (blue) genes in ^AGTR1+^CAFs administered with losartan. CCN1, CCN2, ANKRD1, GLI2 and MFAP5 are indicated with text. (G) qRT‐PCR results show the mRNA level of Hippo pathway targeted genes after administering losartan or valsartan in CAFs #1 and #2. Data are presented as mean ± SD (*n* = 3) and compared by *t*‐test. *p < .05, **p < .01, ***p < .001. ****p < .0001.

**FIGURE 5:**
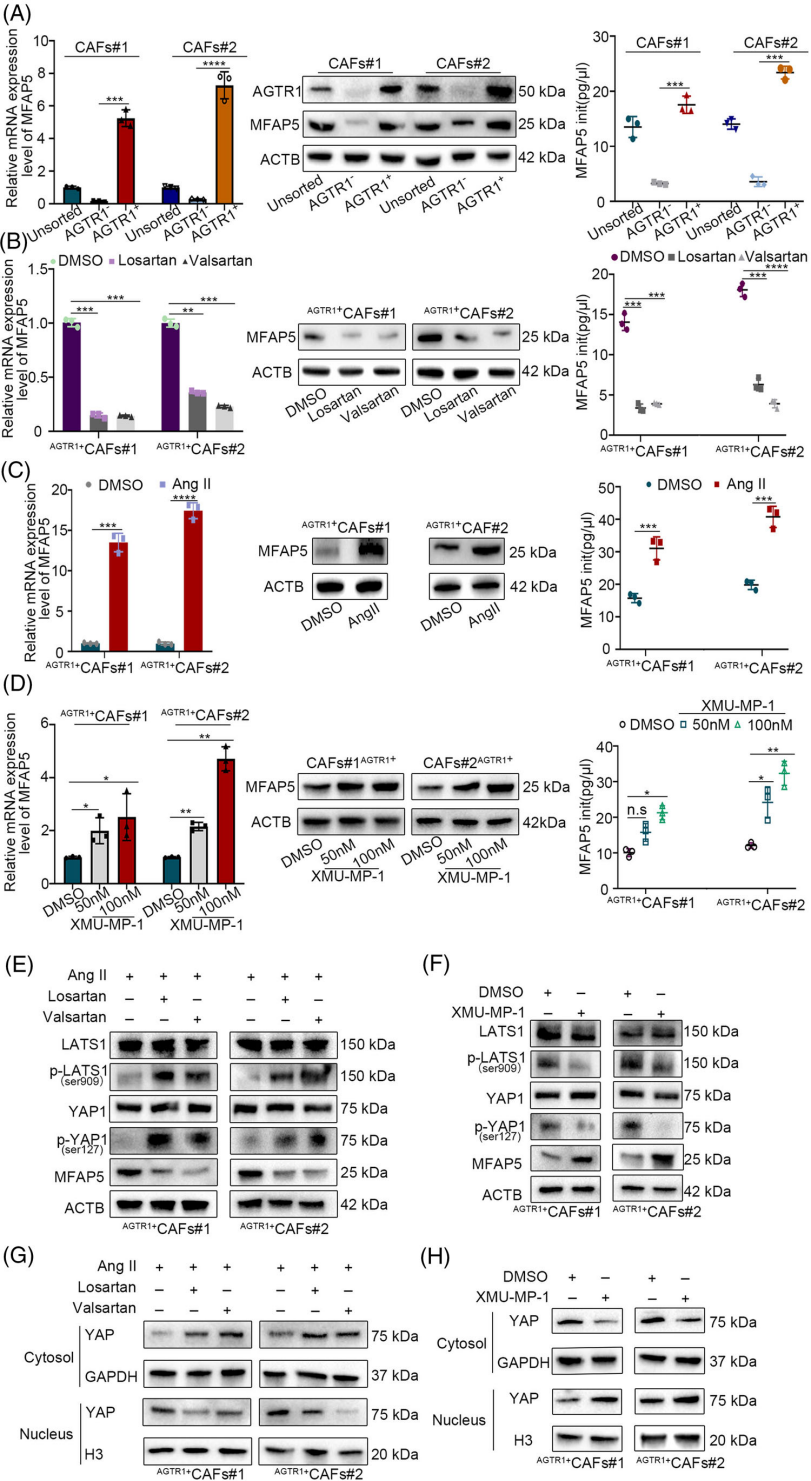
Angiotensin receptor blockers (ARBs) inhibited the secretion of MFAP5 by the Hippo signaling pathway in ^AGTR1+^CAFs (cancer‐associated fibroblasts). (A–D) Western blot and qRT‐PCR results show the expression levels of MFAP5 in CAFs. ELISA results show the secretion level of MFAP5 in CAFs. (E and F) Western blot results show the protein level of LATS1, LATS1 (Ser909), YAP, YAP (Ser127) and MFAP5 in whole‐cell lysates from ^AGTR1+^CAFs #1 and #2 after administrating Ang II (10 nM) with losartan/valsartan/saline or XMU‐MP‐1 (100 nM)/DMSO. (G) Western blot results show the protein levels of YAP in the cytosol and nucleus of ^AGTR1+^CAFs #1 and #2 after administering losartan/valsartan or (H) XMU‐MP‐1/DMSO. GAPDH and H3 were defined as loading control for cytosol and nucleus, respectively. Data are presented as mean ± SD (*n* = 3) and compared by *t*‐test. *p < .05, **p < .01, ***p < .001, ****p < .0001, n.s. = no statistical differences.

To further clarify how ARBs attenuate the viability of ^AGTR1+^CAFs, the transcription differences between losartan‐administered ^AGTR1+^CAFs and DMSO‐administered ^AGTR1+^CAFs were compared using RNA sequencing (Figure 4A,B). The differential gene expression lists have been added to Supporting File S1. The results of the GO analysis indicated that a wide range of transcripts was altered after losartan administration (Figure 4C). The results of the Kyoto Encyclopedia of Genes and Genomes (KEGG) and GSEA analyses showed that the mRNA levels of the Hippo signaling pathway target genes were markedly reduced with losartan administration (Figure 4D,E and Figure S7A,B) and were verified (Figure 4G and Figure S8C). Further GSEA analysis of the NFKB and FOXO pathways was inconsistent with the KEGG results (Figure S8A,B). The volcano plot showed the mRNA levels of the Hippo signaling pathway target genes, which were significantly decreased in losartan‐administered ^AGTR1+^CAFs (Figure 4F). In our previous study, MFAP5, an ECM glycoprotein, was demonstrated to participate in Notch1 signaling activation. This glycoprotein was associated with the prognosis of iCCA and could be identified as a diagnostic index and therapeutic target for iCCA (Figure S15A–E).^16^ By reviewing the MFAP5‐IHC staining of iCCA specimens, we noticed that the positive staining of MFAP5 was abundant in iCCA tissues, especially in the tumour stroma (Figure S9A). Using Western blotting and qRT‐PCR, the expression of MAFP5 was more significant in the CAFs than in tumour cells (Figure S9B,C). The ELISA showed that the medium of CAFs had higher levels of MFAP5 than that of tumour cells (Figure S9D). Interestingly, we found that the mRNA, protein and secretion levels of MFAP5 were significantly higher in ^AGTR1+^CAFs than in ^AGTR1−^CAFs. The mRNA, protein and secretion levels of MFAP5 in ^AGTR1+^CAFs were reduced considerably after the administration of ARBs (Figure 5A,B and Figure S13B,C). Ang II, the ligand of AGTR1, reportedly has a higher concentration in iCCA tissues than in HCC and normal liver tissues.^8^ We found that the mRNA, protein and secretion levels of MFAP5 were markedly increased by Ang II, which has not yet been reported (Figure 5C and Figure S13D). Recently, many studies have demonstrated that molecules secreted by tumour cells and stromal cells (including CAFs) can mediate the interactions between each other.^18^ Therefore, we hypothesized that ARBs might suppress the progression of iCCA by inhibiting the secretion of MFAP5 by ^AGTR1+^CAFs.

### 3.4 ARBs inhibited the secretion of ^AGTR1+^CAFs‐derived MFAP5 by the Hippo signaling pathway

We further investigated the mechanisms underlying ARB regulation of CAF‐derived MFAP5 expression. The RNA‐seq results showed that ARBs could regulate the Hippo signaling pathway. MFAP5 reportedly is one of the target genes of the Hippo signaling pathway. Moreover, scRNA‐seq data (GSE142784) showed that MFAP5 expression was correlated with AGTR1 expression (Figure S9G–J). Therefore, we deduced that ARBs might regulate CAF‐derived MFAP5 via the Hippo signaling pathway. XMU‐MP‐1 is a reversible, potent and selective inhibitor of MST1/2, which is one of the core molecules of the Hippo signaling pathway.^19^ In the presence of XMU‐MP‐1, the mRNA, protein and secretion levels of MFAP5 significantly increased (Figure 5D and Figure S13E). We next assessed the phosphorylation status of LATS1 and YAP and found that neither losartan nor valsartan stimulated their expressions. The ARBs‐administered group exhibited that ^AGTR1+^CAFs attenuated p‐LATS1 and p‐YAP downregulation, thus indicating that ARBs influenced the deactivation of LATS1 and YAP. The expression of MFAP5 (YAP/TEAD target gene) was decreased by ARB administration (Figure 5E and Figure S13F). Further analysis demonstrated that XMU‐MP‐1 effectively promoted MFAP5 expression by inhibiting YAP1 phosphorylation (Figure 5F and Figure S13G). Following its dephosphorylation, YAP was translocated to the nucleus to promote the transcription of target genes. Western blotting was used to evaluate the protein levels of YAP in the cytoplasmic and nuclear fractions of ^AGTR1+^CAFs. Upon ARB administration, YAP protein levels increased in the cytoplasm and decreased in the nucleus. Ang II‐induced alterations were reversed by ARB administration (Figure 5G and Figure S13H). Conversely, XMU‐MP‐1 administration resulted in increased levels of YAP1 dephosphorylation (Figure 5F), increased nuclear YAP levels and decreased cytoplasmic YAP levels in ^AGTR1+^CAFs compared with DMSO controls (Figure 5H and Figure S13I). These results demonstrate that ARBs inhibit the secretion of ^AGTR1+^CAF‐derived MFAP5 via the Hippo signaling pathway.

### 3.5 CAFs‐derived MFAP5 promoted proliferation of iCCA cells in vitro and in vivo

**FIGURE 6:**
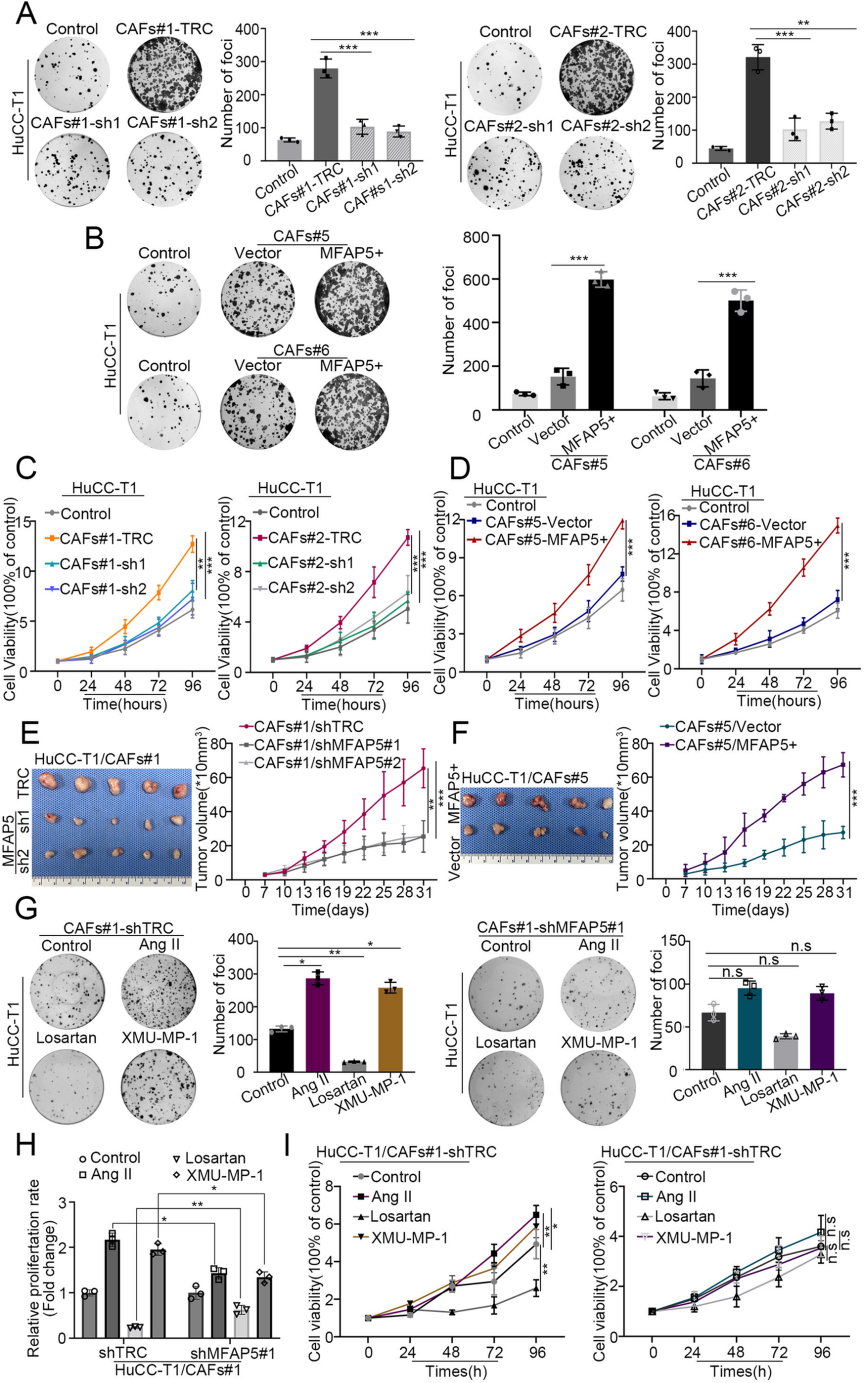
Cancer‐associated fibroblasts (CAFs)‐derived MFAP5 promoted the proliferation ability of intrahepatic cholangiocarcinoma (iCCA) cells. (A and B) Colony formation results show the colony‐forming ability of HuCC‐T1 (T1) cells co‐cultured with conditional medium obtained from CAFs (Figure S10A,B). The histogram shows the number of foci in T1 cells co‐cultured with conditional medium obtained from the respective groups (control refers to the group with the T1 cell alone; statistical analysis was performed by *t*‐test of the shMFAP5/MFAP5+ versus the TRC/Vector group). (C and D) Cell viability results show the proliferation rate of HuCC‐T1 cells co‐cultured with conditional medium obtained from CAFs in Figure S10A,B. The line plot shows cell viability in T1 cells co‐cultured with conditional medium obtained from the respective groups (control refers to the group with the T1 cell alone; statistical analysis was performed by *t*‐test of the shMFAP5/MFAP5+ vs. the TRC/Vector group). (E and F) Tumour growth curves show the growth rate of xenograft tumours co‐injected of HuCC‐T1 and CAFs (*n* = 5). (G) Colony formation and (I) cell viability shows the colony forming and proliferation abilities of HuCC‐T1 cells co‐cultured with CAFs #1‐shTRC and CAFs #1‐shMFAP5#1 (control refers to T1 cell co‐cultured with conditional medium obtained from CAFs #1‐shTRC and CAFs #1‐shMFAP5#1). (H) The relative proliferation rate of colony formation with Ang II, losartan or XMU‐MP‐1 administration in the respective groups. Data are presented as mean ± SD (*n* = 3) and compared by *t*‐test. *p < .05, **p < .01, ***p < .001, n.s. = no statistical differences.

To elucidate whether CAF‐derived MFAP5 could promote the proliferation of iCCA cells, HuCC‐T1 cells were cultured in a conditioned medium obtained from iCCA‐CAFs. First, we constructed stable MFAP5 knockdown in CAFs #1 and CAFs #2 (with relatively higher expression of MFAP5) (Figure S9C) with shMFAP5 #1 and shMFAP5 #2 (Figure S10A). The ELISA results showed that the concentration of MFAP5 protein was significantly decreased in the supernatant of MFAP5 knockdown CAFs (CAFs #1‐sh1, CAFs #1‐sh2, CAFs #2‐sh1 and CAFs #2‐sh2). A colony formation assay was performed by seeding HuCC‐T1 cells in the lower chamber and CAFs in the upper chamber. The results showed that the colony number of HuCC‐T1 cells, with conditioned medium from MFAP5 knockdown CAFs, was significantly lower than that of the control groups (CAFs #1‐TRC, CAFs #2‐TRC) (Figure 6A). The viability of HuCC‐T1 cells treated with this conditioned medium was significantly inhibited compared to that of the control groups (Figure 6C). We also established stable MFAP5 overexpressed CAFs #5 and #6 (with relatively decreased MFAP5 expression) (Figure S10B). The colony‐forming ability and cell viability were markedly promoted by MFAP5 overexpressed CAFs in HuCC‐T1 cells (Figure 6B,D). We co‐cultured HuCC‐T1 cells with CAFs in vivo. The results showed that the tumour growth rates of the MFAP5 knockdown CAFs groups were significantly slower than that of the control (TRC) groups (Figure 6E). In the MFAP5‐overexpressing CAFs group, the tumour growth rate was markedly higher than that in the control (Vector) group (Figure 6F). To confirm that ARBs inhibit the progression of iCCA cells through AGTR1/Hippo/MFAP5, we treated MFAP5 knockdown/control CAFs with ARBs/Ang II/XMU‐MP‐1, and extracted the supernatant for conditioned cultivation of HuCC‐T1. The results showed that the colony‐forming ability and cell viability of HuCC‐T1 cells cultured in a conditioned medium (from MFAP5 knockdown CAFs) did not significantly respond to ARBs/Ang II/XMU‐MP‐1 compared with the control. These results indicated that MFAP5 knockdown CAFs were not sensitive to ARBs/Ang II/XMU‐MP‐1 compared to control CAFs (Figure 6G–I), confirming that ARBs inhibited the progression of iCCA cells through AGTR1/Hippo/MFAP5. These results demonstrated that CAF‐derived MFAP5 promoted the progression of iCCA cells.

We aimed to investigate the molecular mechanism by which MFAP5 promotes iCCA cell progression. Significant changes in chromatin structure with peaks were observed in recombinant protein MFAP5‐treated (recMFAP5) iCCA cells compared to DMSO‐treated iCCA cells in our previous study.^16^ The list of enriched genes has been added to Supporting File S2. The Notch1 signaling pathway was one of many that changed significantly (Figure S11A–C). The genes assigned to the canonical WNT signaling pathway were also upregulated considerably in iCCA cells treated with recMFAP5 (Figure S11D). Figure S11G shows the promoter region changes in these core genes, including SOX17, TCF7L1, FGFR2, CTNNB1 and MLLT3. However, we had to explore the connection between MFAP5 and the canonical WNT signaling pathway. To verify whether MFAP5 promotes iCCA cell proliferation via the Notch1 signaling pathway, we tested the effect of Notch1 inhibitors (FLI‐06 and crenigacestat) on recMFAP5‐induced cells. The increased proliferation of iCCA cells induced by recMFAP5 was markedly inhibited in the presence of FLI‐06 or crenigacestat (Figure S11E). Western blot analysis revealed that FLI‐06 and crenigacestat effectively reversed MFAP5‐induced Notch1 signaling pathway activation (Figure S11F). Moreover, to confirm that CAFs act on tumour cells via ligand MFAP5, we analyzed the single‐cell RNA‐seq dataset GSE138709 by using the NicheNET method.^20^ We performed cell–cell interaction analysis with fibroblasts as sender and epithelial cells as receiver (Figure S12A,B). Results showed that the high value of regulatory potential between MFAP5 and some of its top predicted NOTCH1/WNT pathway target genes (CCND1, HES1, MYC, RBPJ, NOTCH1, CCN3, CTNNB1 and SOX17) in inferring signaling paths in tumour tissues (Figure S12C,D). But MFAP5 was not an active ligand in tumour‐adjacent tissues (Figure S12E,F). These results indicated that there is existing cell–cell communication between CAFs and tumour cells by MFAP5. The MFAP5 in the TME activates the Notch1 pathway and boosts the transcription of downstream genes, thus promoting the proliferation of iCCA cells.

## 4 DISCUSSION

Hypertension is a common comorbidity in patients with cancer and is a risk factor for various cancers. The RAS system, particularly Ang II, plays a key role as a ‘gatekeeper’ in controlling blood pressure. Of note, Okamoto et al. revealed that the Ang II/AGTR1 axis strongly correlated with the activation of HSCs, tumour progression and fibrogenesis of iCCAs.^8^ However, retrospective studies on the prognosis of iCCA remain scarce even in patients with CCA receiving anti‐hypertensive drugs. Our study found benefits to both DFS and OS in the group of iCCA patients on regular anti‐hypertensive drugs, with the advanced pronounced benefits in the ARBs administration to iCCA (pT1a‐3N0M0) patients. In several studies, ARBs have antagonistic effects on tumour growth and angiogenesis.^21^, ^22^ ARBs reportedly increase drug delivery to pancreatic ductal adenocarcinoma (PDAC), thus improving its effect.^23^ The effect of losartan in combination with gemcitabine was investigated in PDAC, and the results showed that losartan increased the DNA incorporation of gemcitabine and enhanced its efficacy (NCT01276613).^24^ Another phase 2 trial revealed that losartan, neoadjuvant therapy with FOLFIRINOX, and chemoradiotherapy contributed to the downstaging of locally advanced PDAC, resulting in an R0 resection rate of 61% (NCT01821729).^25^ It could be observed that ARBs play an important role in anti‐solid tumour progression. Nevertheless, the interactions between iCCA and ARBs remain largely unknown. Therefore, we aimed to investigate the mechanism of action of ARBs that interferes with iCCA progression. Through quantitative experiments, we found that AGTR1, the target receptor of ARBs, was expressed in both iCCA‐CAFs and tumour cells. Tumours of iCCA are hard and solid, 80%–90% of which comprise CAFs and ECM.^26^ CAFs have been considered co‐conspirators of tumour cells that promote tumour growth.^27^ We harvested CAFs from fresh iCCA tissues by cell sorting using immunomagnetic beads, and found that the effect of ARBs on CAFs seems influenced by AGTR1 expression. Interestingly, GO enrichment analysis was implemented with marker genes of AGTR1+ fibroblasts and AGTR1− fibroblast subclusters in the dataset GSE142784. The results showed significant functional differences between these two subclusters of fibroblasts, with the AGTR1+ subcluster being significantly enriched for ECM organization (Figure S9K). In contrast, the AGTR1− subcluster was more associated with the altered function of leukocytes (Figure S9L). These findings corroborated the high heterogeneity of iCCA‐CAFs. After sorting iCCA‐CAFs into ^AGTR1+^CAFs and ^AGTR1−^CAFs, we found that ARBs significantly reduced the viability of ^AGTR1+^CAFs in vitro, which was verified by xenograft assays in vivo. An RNA‐seq assay of AGTR1+CAFs #1 and #2 after administration of losartan, a representative ARB drug, was performed to elucidate the molecular mechanism. Based on these results, we speculated that ARBs are involved in the regulation of Hippo signaling pathway. Several Hippo signaling pathway targets (CCN1, CCN2, ANKRD1, MFAP5) were significantly decreased in ARB‐administered ^AGTR1+^CAFs compared to DMSO‐treated ^AGTR1+^CAFs. Interestingly, MFAP5, a glycoprotein with an important biological function in the TME, was also found to be an important molecule in our previous study.^16^ However, we did not know whether it was secreted by CAFs. In recent years, many studies have shown that CAF‐derived MFAP5 plays a paracrine role in the progression of various cancers. CAF‐derived MFAP5 upregulates the lipoma‐preferred partner (LPP) gene to improve the efficacy of chemotherapy in ovarian cancer.^28^ In breast cancer, CAF‐derived MFAP5 affects the invasion and migration of tumour cells through the Notch1/slug pathway.^29^ In oral tongue squamous cell carcinoma, CAF‐derived MFAP5 plays a paracrine role in cell growth and migration via activation of the MAPK and AKT pathways.^30^ In CCA cells, the expression and secretion of MFAP5 are regulated by intranuclear translocation of the YAP/TEAD transcription factor.^31^ Saikawa et al. found that losartan interfered with YAP nuclear shuttling in CCA cells.^32^ However, the effect of ARBs on CAF‐derived MFAP5 secretion remains unknown.

In the present study, we found that the administration of ARBs in ^AGTR1+^CAFs significantly decreased the mRNA, protein and secretion levels of MFAP5. The ELISA showed that CAFs secreted higher levels of MFAP5 than HuCC‐T1 cells. This finding prompted us to verify the role of CAF‐derived MFAP5 in iCCA cell proliferation. In our previous study, recMFAP5 was co‐cultured with purified recombinant MFAP5 in iCCA cell proliferation. In this study, we evaluated the function of CAF‐derived MFAP5 in iCCA using a conditioned medium. Consistent with the effects of recMFAP5, CAF‐derived MFAP5 promoted the proliferation of iCCA cells in vitro and in vivo. The Hippo pathway is activated by stromal stiffness in solid tumour tissues, and increasing evidence has shown that YAP is activated in CAFs.^33^ YAP controls the expression of several ECM factors, including diaphanous homolog 3 (DIAPH3), connective tissue growth factor (CTGF) and myosin light chain‐2 (MLC).^34^ MFAP5 is an ECM glycoprotein mainly distributed in the stroma and regulated by YAP/TAED in CCA cells. However, whether YAP is involved in regulating MFAP5 in CAFs has not yet been reported. Our results showed that the dephosphorylation of cytoplasmic YAP promoted intranuclear translocation. Subsequently, the transcription of MFAP5 was initiated in CAFs. Further results showed that ARBs effectively attenuated the CAF‐derived MFAP5 expression. We identified a regulatory effect of Ang II/AGTR1 on the signaling axis of LATS1/YAP in ^AGTR1+^CAFs.

**FIGURE 7:**
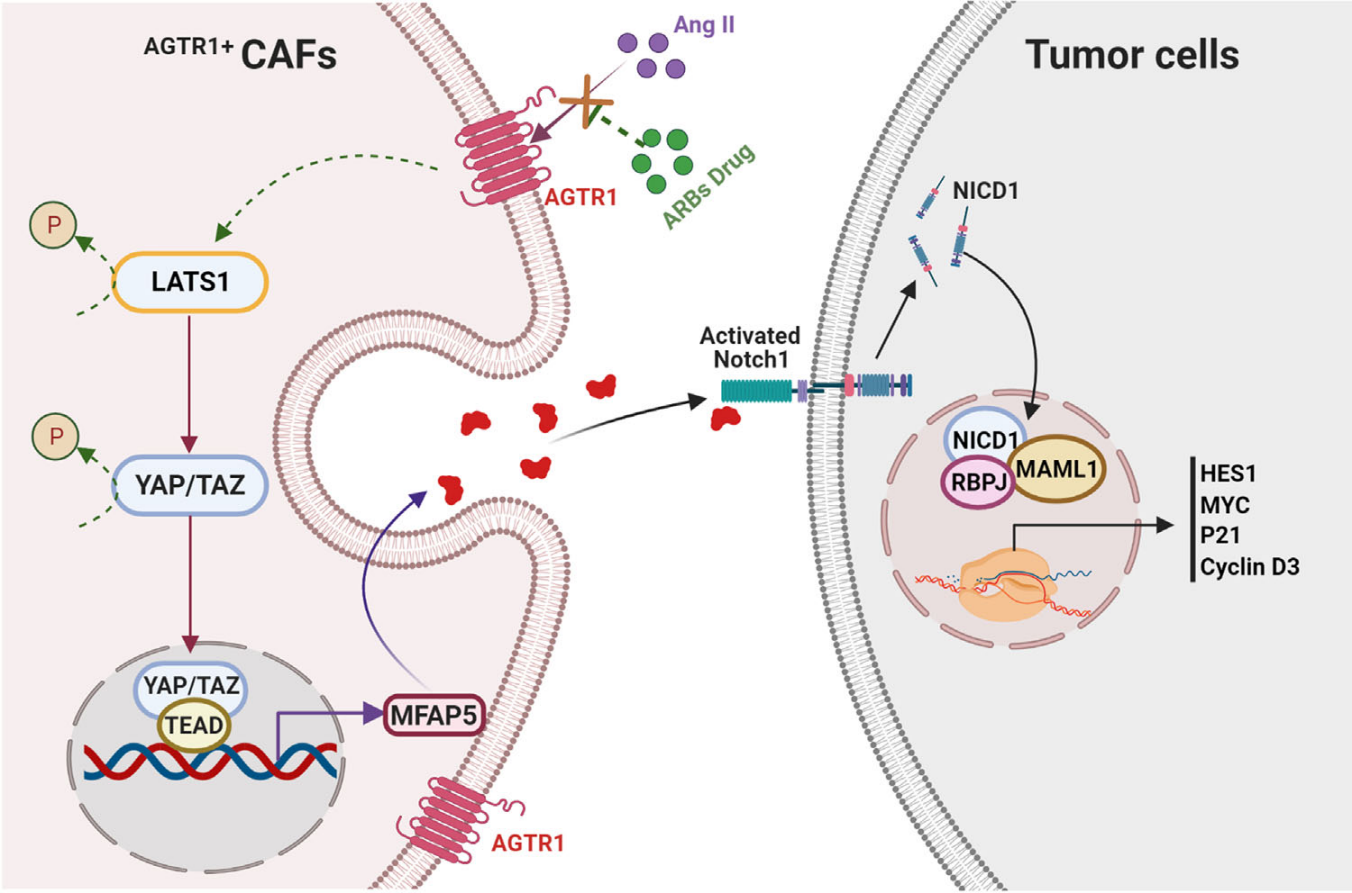
Proposed schematic models illustrating that angiotensin receptor blockers (ARBs) inhibit the viability of ^AGTR1+^CAFs (cancer‐associated fibroblasts), decrease the secretion of ^AGTR1+^CAFs‐derived MFAP5 by the Hippo pathway, weaken the interaction between CAFs and intrahepatic cholangiocarcinoma (iCCA) cells, and retard the proliferation of iCCA cells by Notch1 pathway.

In conclusion, our results revealed that ARB administration was beneficial for the prognosis of patients with iCCA. ARBs inhibited the secretion of ^AGTR1+^CAF‐derived MFAP5 via the Hippo pathway. The secreted MFAP5 regulated the activation of the Notch1 pathway and mediated the proliferation ability of the iCCA cells (Figure 7). Hence, ARBs can potentially be novel options for treating patients with iCCA.

## CONFLICT OF INTEREST STATEMENT

The authors declare no conflicts of interest.

## Supporting information

Supporting Information

Supporting Information

Supporting Information
