# Supplementary material for: Angiotensin receptor blockers retard the progression and fibrosis via inhibiting the viability of AGTR1+CAFs in intrahepatic cholangiocarcinoma
Source: Clin Transl Med. 2023 Feb 28;13(3):e1213. doi: 10.1002/ctm2.1213 (PMC9975461; doi:10.1002/ctm2.1213)
Supplement: Supplementary file 3 — Supporting Information [file CTM2-13-e1213-s002.docx]

**Supplementary Information**

**Title:** **Angiotensin receptor blockers retard the progression and fibrosis via inhibiting the viability of ^AGTR1+^CAFs in intrahepatic cholangiocarcinoma**

**Running title：ARBs attenuate the aggressiveness of iCCA**

Jian-Hui Li*, 1, M.D., Xiao Wu *, 1, M.D., Xuhao Ni*, 1, Ph.D., Ya-Xiong Li, 1, M.D., Ph.D., Long Xu, 3, M.D, Xiao-Yi Hao, 4, M.D., Ph.D., Wei Zhao ^#^, 2, M.D., Ph.D., Xiao-Xu Zhu ^#^, 1, Ph.D., Xiao-Yu Yin^#^, 1, M.D., Ph.D.

**Contents**

**Supplementary Figure 1**

**Supplementary Figure 2**

**Supplementary Figure 3**

**Supplementary Figure 4**

**Supplementary Figure 5**

**Supplementary Figure 6**

**Supplementary Figure 7**

**Supplementary Figure 8**

**Supplementary Figure 9**

**Supplementary Figure 10**

**Supplementary Figure 11**

**Supplementary Figure 12**

**Supplementary Figure 13**

**Supplementary Figure 14**

**Supplementary Figure 15**

**Supplementary Table 1-3**

**Supplementary Methods**

**Supplementary Figure 1. iCCA patients benefited from RAS inhibitors administration**

(A) Flow diagram of patients who were assessed for eligibility for the study. (B) Survival analysis based on RAS inhibitors usage in iCCA patients (n=34, RAS (-) and n=37, RAS (+)). (C, D) Dot plot showed the p-value of logrank in DFS (C) or OS (D) corresponds to the expression of AGTR1(FPKM). (E, F) Prognostic results based on AGTR1 expression in iCCA tissues (the protein level of AGTR1, split at median of IHC score, score＞6, n=51 and score ≤6, n=51). (G) HE and Masson trichrome staining of iCCA and paracancerous tissue (scale bar=250μm). (H) qRT-PCR results showed the mRNA level of AGTR1 in CAFs (#1-#6) and tumor cells (TCs) (RBE+HuCC-T1) (related to Figure 1G). (I) The AGTR1 expression level detected in primary CAFs and tumor cells (case#1, #2, #4, #5) by qRT-PCR assay. Data were presented as mean ± SD and compared by t-test. **p*＜.05, ***p*＜.01, ****p*＜.001,*****p*＜.0001.

**
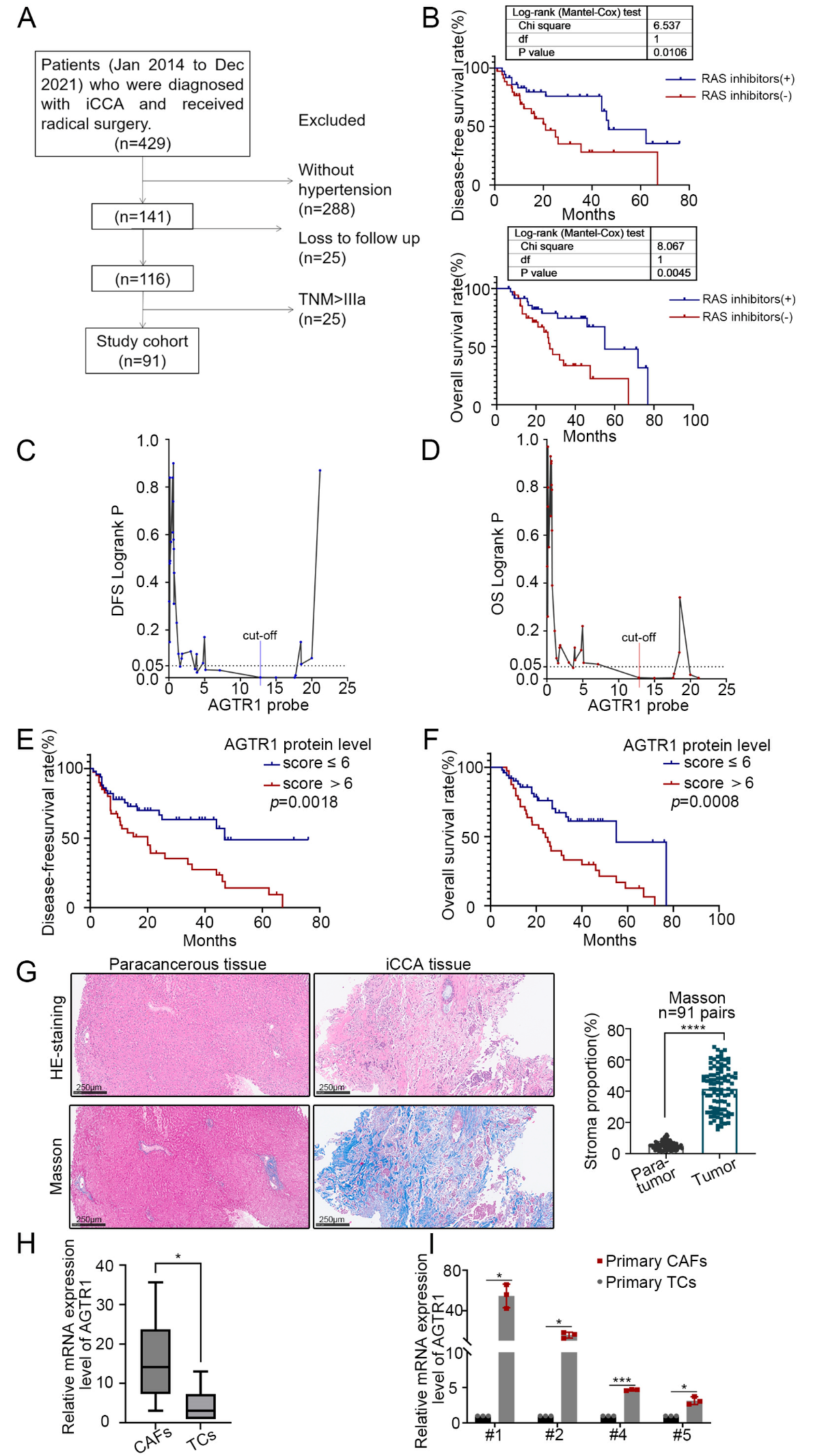
**

**Supplementary Figure 2. The purity of the fibroblasts was validated by immunofluorescence staining**

(A) The microscopic morphology of CAFS#1-#6 (scale bar=100μm). (B, C) Immunofluorescence staining of CAFs#1 and CAFs#2 (α-SMA: green; DAPI: blue, scale bar=100μm). The histogram counts the number of cells in 100x microscopic view (n=5).

**
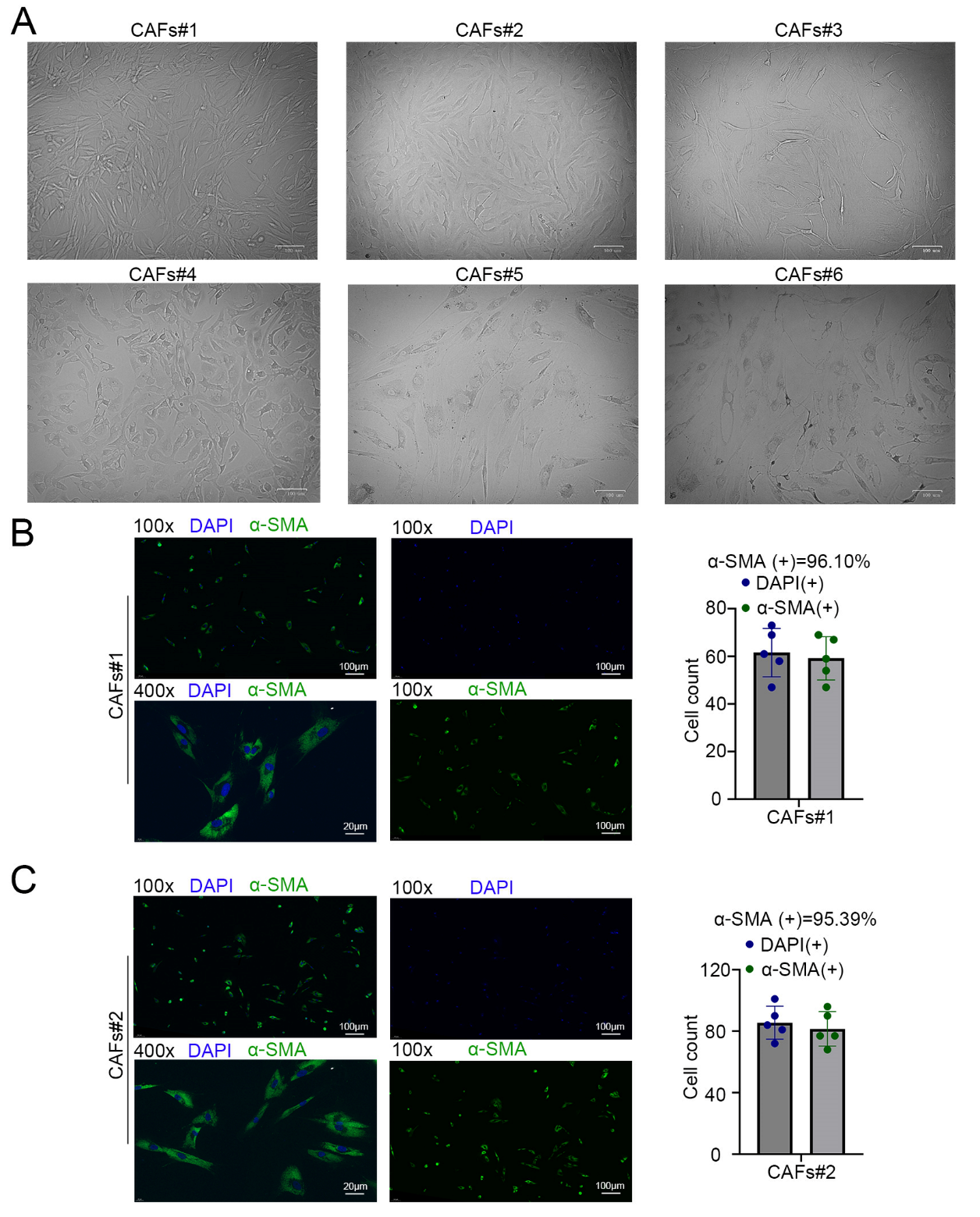
**

**Supplementary Figure 3. The expression of AGTR1 was generally higher in fibroblasts than in epithelial cells in public scRNA-seq data (GSE138709)**

(A) tSNE plots for the cell type identification of 4946 high-quality single cells. (B) Dot plot showed that the visualization of AGTR1 in each cluster (GSE138709). (Dot size reflected the percentage of cells in a cluster expressing AGTR1; dot color reflected the expression level). (C) tSNE plot showed the expression level of AGTR1 in each cluster. (D) tSNE plots for the cell type identification of 4946 high-quality single cells after cell type annotation of single cells. (E) Heatmap showed the expression level of AGTR1 in epithelial cells and fibroblasts (samples with a TPM value of 0 were excluded). (F) Histogram showed the AGTR1 probe (TPM) in epithelial cells and fibroblasts. (G, I) Cell viability results showed the different proliferation rates after administrating losartan (300μM) or valsartan (100μM) in CAFs#3, #4. (H, J) Gel contraction results of CAFs with losartan or valsartan administration at different time points. Left panel: contracted gel within black, green, purple line represented DMSO, losartan, and valsartan, respectively. Right panel: quantification of gel contraction rate under losartan and valsartan. Data were presented as mean ± SD (n=3) and compared by t-test. **p*<.05, ***p*<.01, ****p*<.001, *****p*<.0001, n.s=no statistical differences.

**
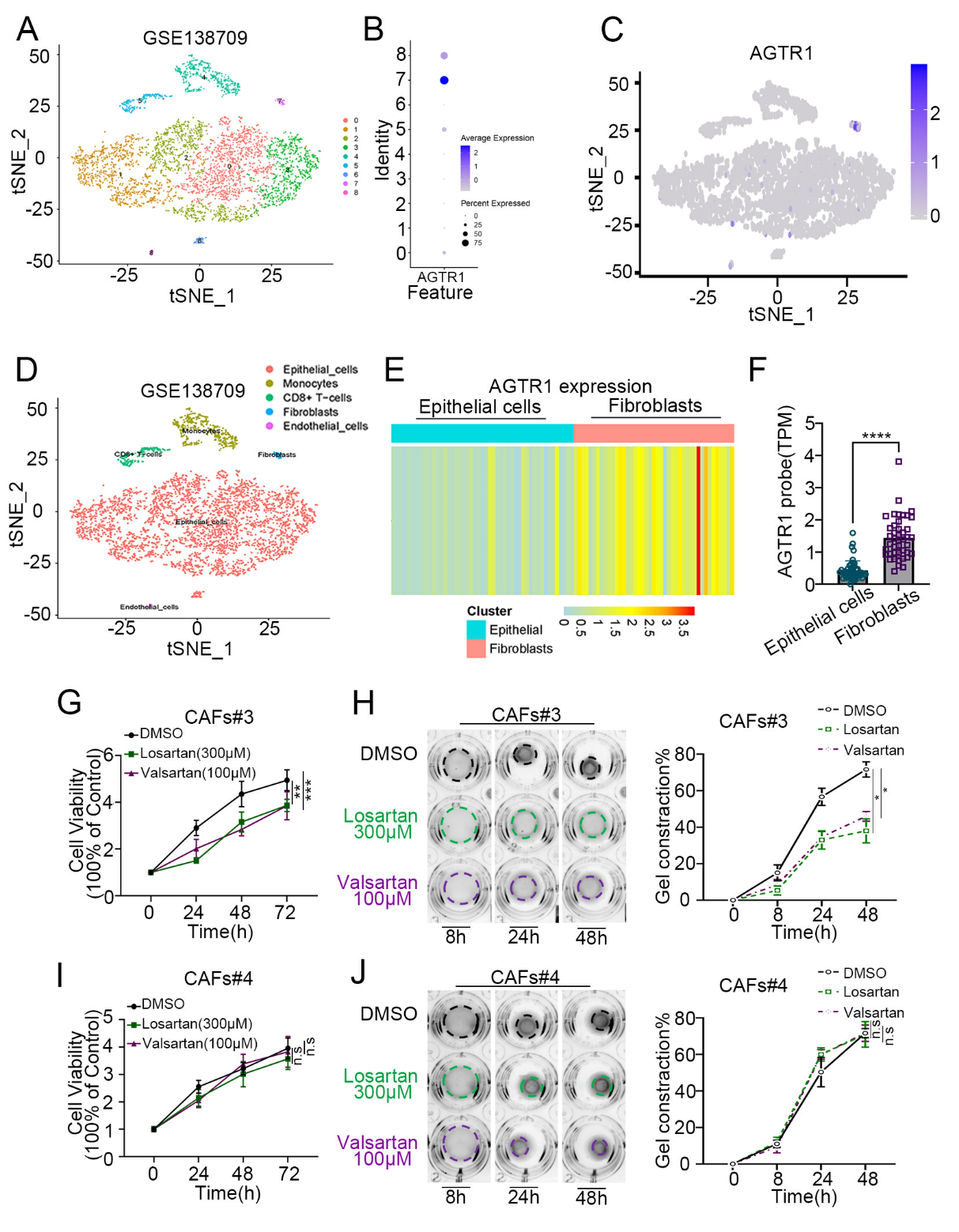
**

**Supplementary Figure 4. Strategy for analyzing the percentage of ^AGTR1+^CAFs of primary unsorted CAFs via flow cytometry**

(A) Strategy for analyzing the percentage of ^AGTR1+^CAFs of primary unsorted CAFs (#1-#6) via flow cytometry. (B) The correlation of AGTR1 positive rates (FACS) and AGTR1 expression level (immunoblot) was shown with point plot, analyzed by Pearson’s correlation.


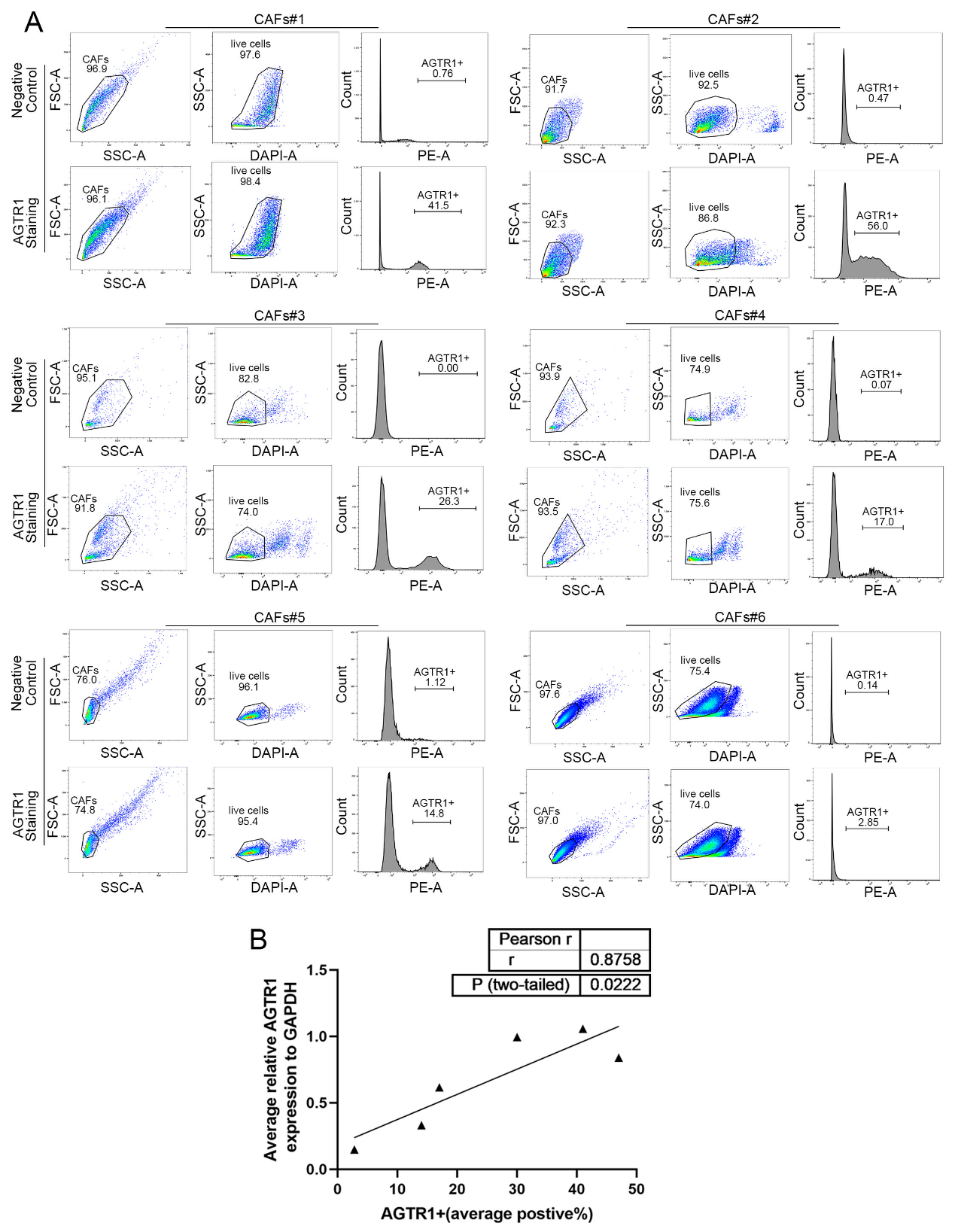


**Supplementary Figure 5. ARBs attenuated the viability of ^AGTR1+^CAFs**

(A, C) Cell viability results showed the different proliferation rate after administrating losartan (300μM) or valsartan (100μM) in ^AGTR1+^CAFs#1, #2. (B, D) Gel contraction results of 1-fold or 5-fold cells of ^AGTR1+^CAFs#1, #2 with losartan or valsartan administration at different time points. Left panel: contracted gel within green, purple line represented losartan and valsartan, respectively. Right panel: quantification of gel contraction rate under losartan and valsartan. (E) Tumor growth curves showed the growth rate of xenograft tumors co-injected of HuCC-T1 and CAFs. (F) Tumor growth curves showed the growth rate of xenograft tumors co-injected of RBE and CAFs. (G) The relative tumor volume of xenograft tumors with saline or losartan administration in respective groups. Data were presented as mean ± SD and compared by t-test. **p*＜.05,*****p*＜.0001, n.s=no statistical differences.

**
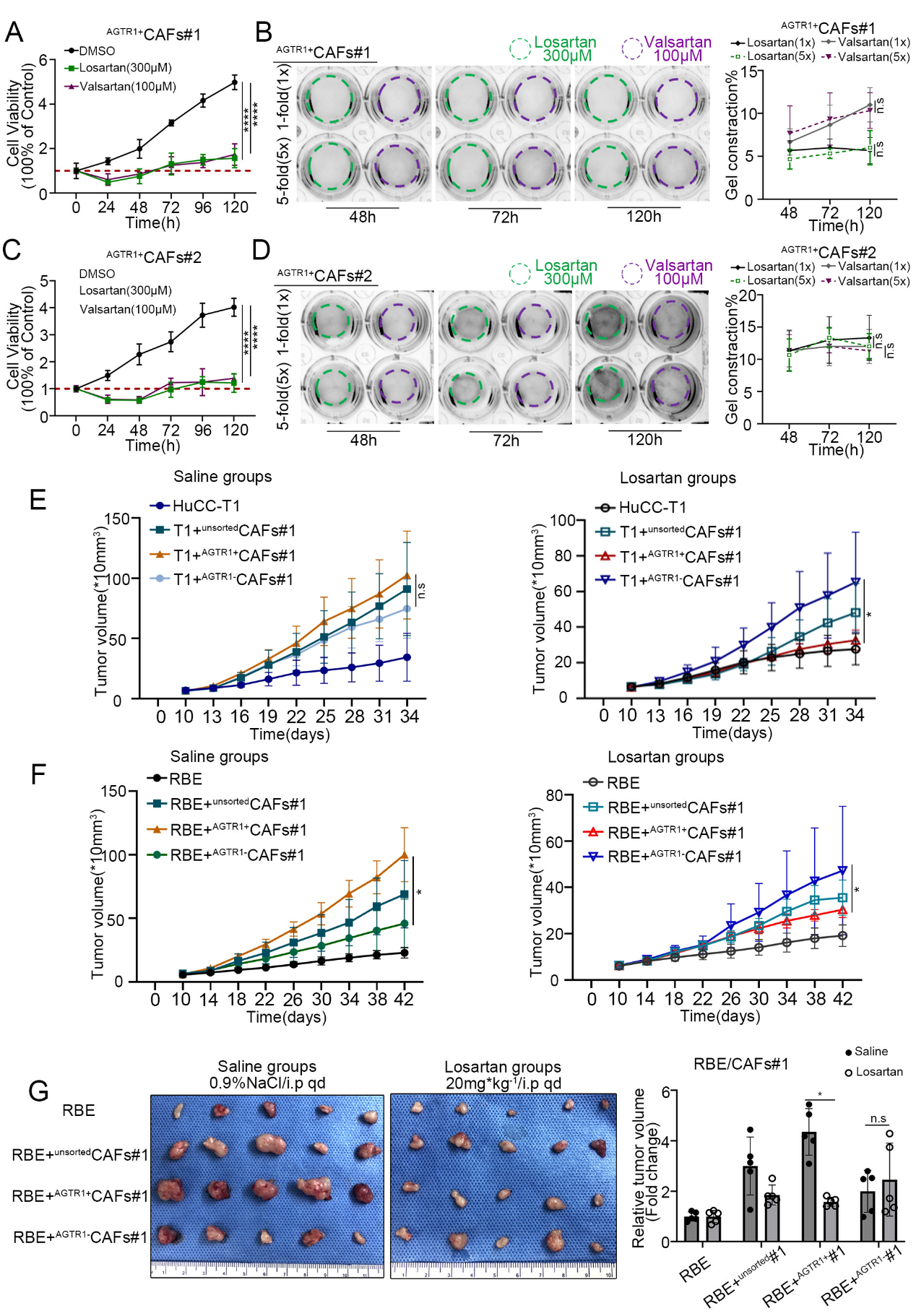
**

**Supplementary Figure 6. The population of ^AGTR1+^CAF was retained in the tumor microenvironment and correlated with increased matrix deposition**

(A, C) Masson trichrome staining of subcutaneous tumor samples. The histogram plot showed the stroma proportion of the subcutaneous tumors (n=5). (B) Strategy for analyzing the percentage of ^AGTR1+^CAFs of unsorted CAFs from subcutaneous tumors via flow cytometry. The histogram showed the percentage of ^AGTR1+^CAFs in saline and losartan groups (n=3). Data were presented as mean ± SD and compared by t-test. ****p*<.001, *****p*<.0001.


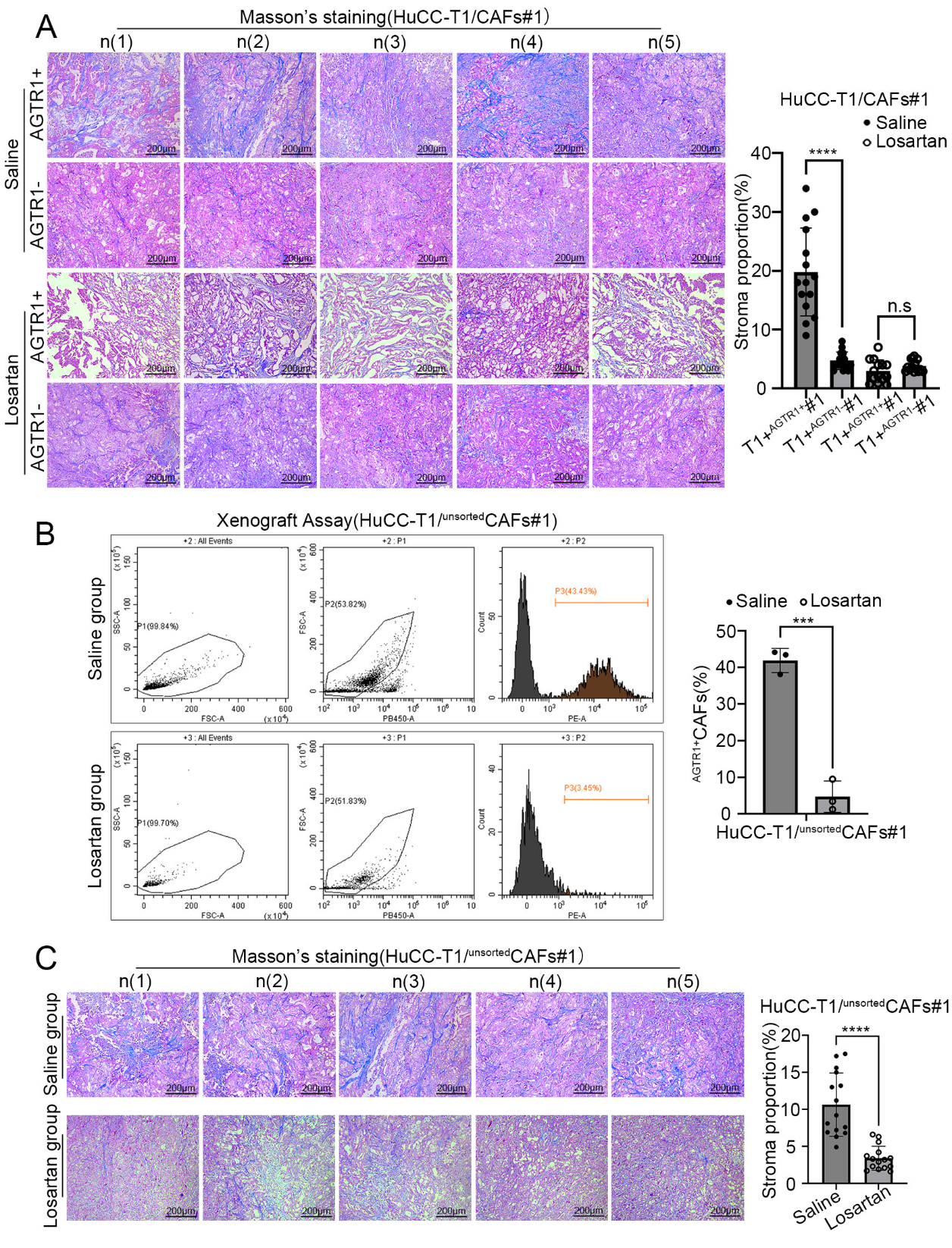


**Supplementary Figure 7. Hippo signaling pathway target genes were markedly reduced with losartan administration**

(A) KEGG pathway annotation using genes which were differentially expressed were defined as significant in losartan group versus DMSO group (pathway id: map04390, (green: down-regulated, red: up-regulated)). (B) Histogram showed DEGs number of the enriched pathway (blue: down-regulated, red: up-regulated).

**

**

**Supplementary Figure 8. The** **GSEA analysis of NFKB and FOXO pathway was not completely consistent with the results of KEGG**

(A, B) GSEA results showed down-regulation of NFKB pathway (A) or FOXO pathway (B) in CAFs administrated with losartan, compared with DMSO. (C) qRT-PCR results showed the mRNA level of Hippo pathway target genes after administrated with losartan or valsartan in CAFs#3, #4. Data were presented as mean ± SD and compared by t-test. **p*＜.05, ***p*＜.01, ****p*<.001.

**
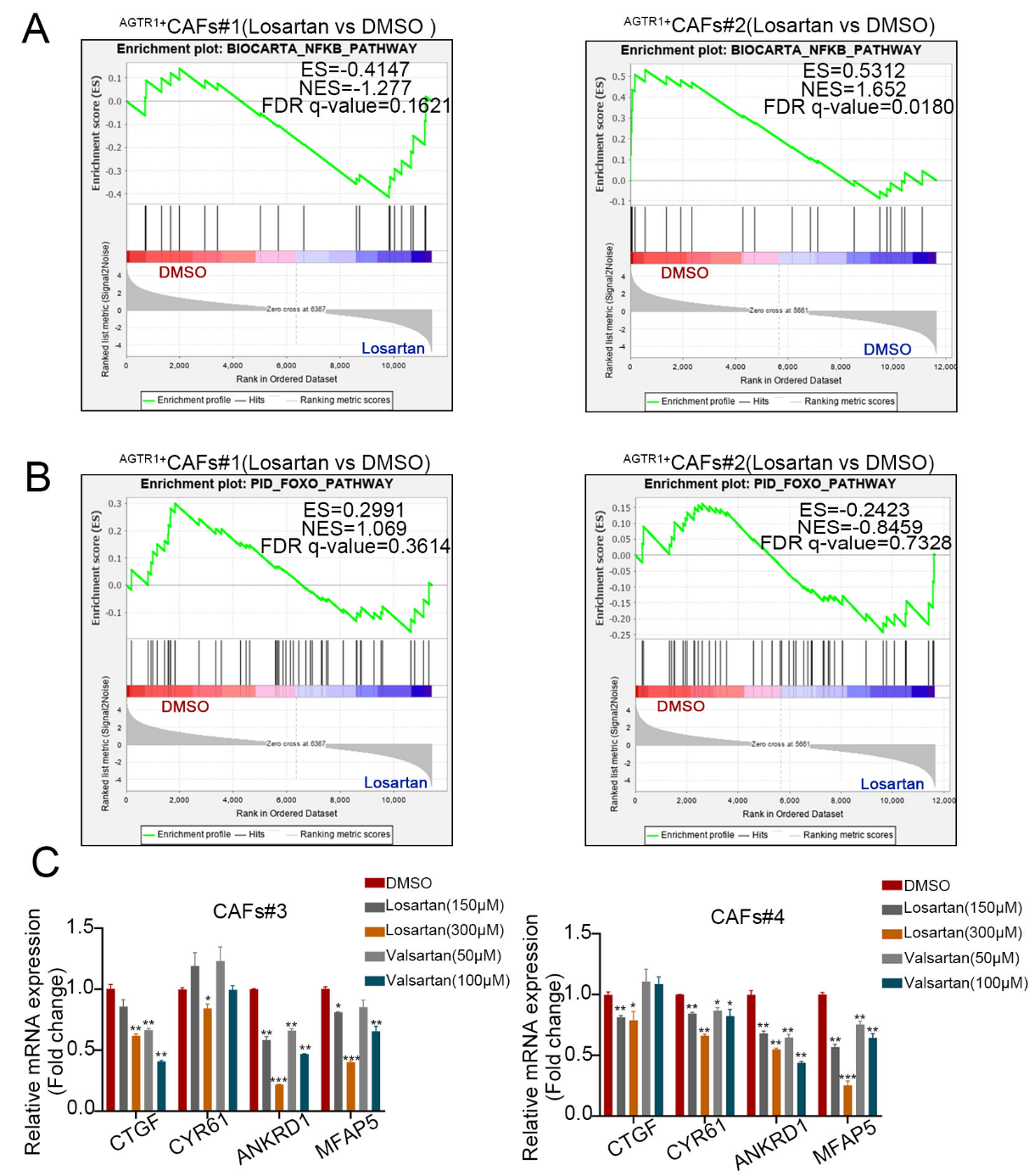
**

**Supplementary Figure 9. CAFs expressed higher level of MFAP5 than iCCA cells**

(A) IHC results showed that MFAP5 protein was enriched in iCCA stroma. (B, C, D) qRT-PCR (B) and western blot (C) results showed the expression level of MFAP5 in CAFs#1-#6, REB, HuCC-T1 and normal fibroblasts#1 (NF-1). ELISA (D) results showed the secretion level of MFAP5 in CAFs#1-#6, REB, HuCC-T1 and normal fibroblasts#1 (NF-1). (E) tSNE plots for the cell type identification of 5292 high-quality single fibroblast cells. (F) Dot plot showed that the visualization of AGTR1/MFAP5 in each cluster (GSE142784). (Dot size reflected the percentage of cells in a cluster expressing AGTR1; dot color reflected the expression level). (G) tSNE plot showed the ^AGTR1+/-^CAFs cluster. (H) tSNE plot showed the expression of MFAP5 in each cluster. (I) Histogram showed the MFAP5 probe (TPM) in ^AGTR1+^CAFs (cluster 0,3,5) and ^AGTR1-^CAFs (cluster 1,2,4,6). (J) The correlation of AGTR1 probe (TPM) and MFAP5 probe (TPM) was shown with point plot, analyzed by Pearson’s correlation. (K, L) GO analysis of marker genes in AGTR1+fibroblast (K) and AGTR1-fibroblast (L) subclusters. Data were presented as mean ± SD and compared by t-test. *****p*<.0001.

**
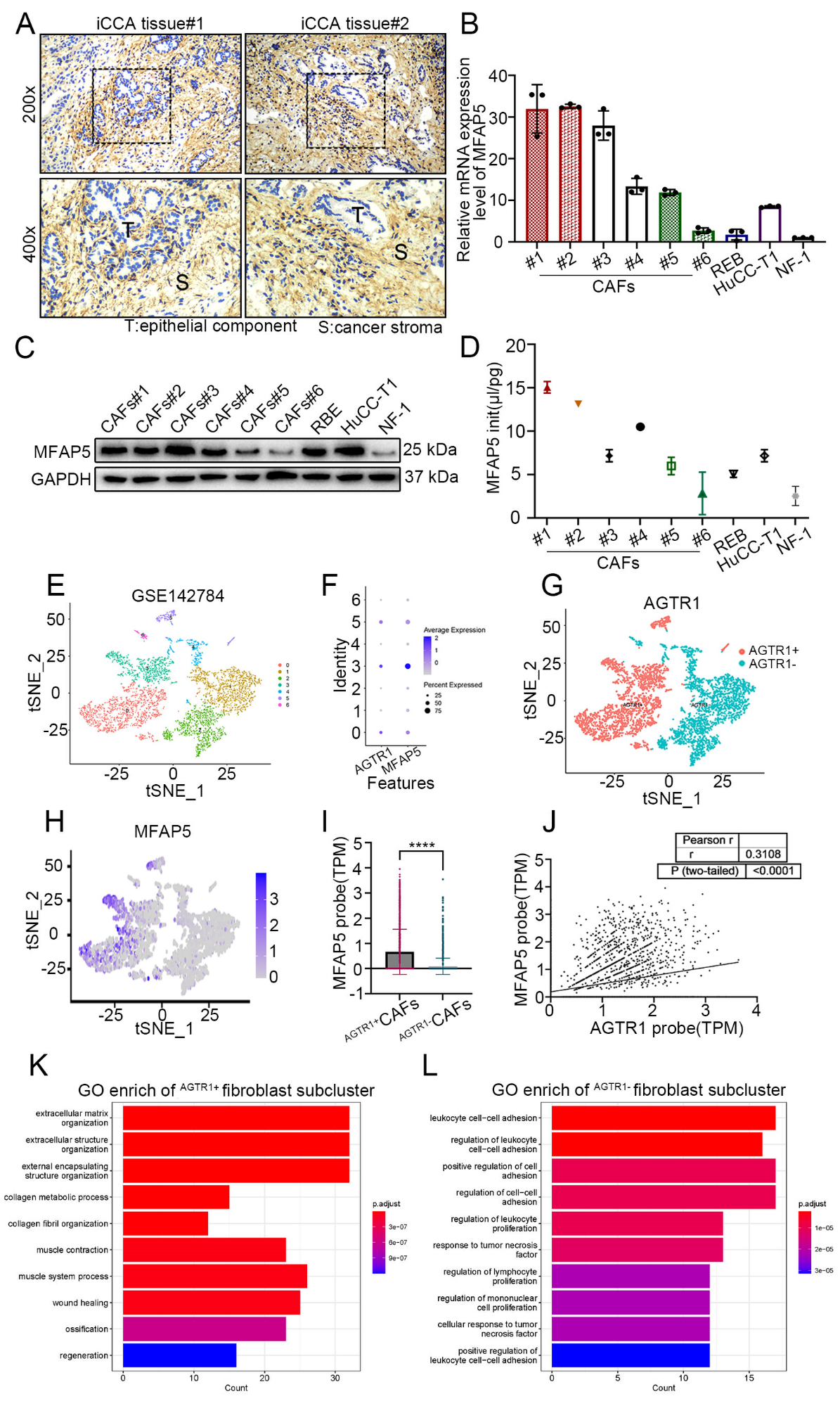
**

**Supplementary Figure 10. The CAFs cell lines of silencing and over-expressed MFAP5 were established**

(A, B) After transfected MFAP5 shRNA (A) or plvx-MFAP5 plasmid (B) in CAFs. Western blot and qRT-PCR results showed the expression level of MFAP5 in CAFs. ELISA results showed the secretion level of MFAP5 in CAFs. Data were presented as mean ± SD and compared by t-test. **p*＜.05, ***p*＜.01,****p*＜.001, *****p*< .0001


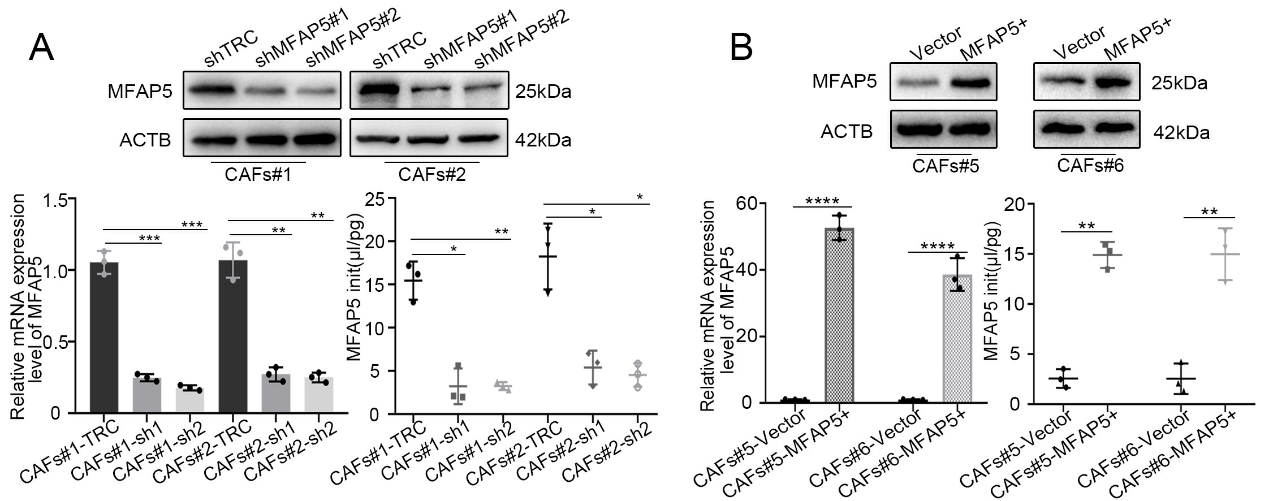


**Supplementary Figure 11. MFAP5 mediated the progression ability of iCCA cells through Notch1 signaling pathway**

**(A)**ATAC-seq heatmap results showed the appearing peaks status in DMSO and recMFAP5 groups. (B) GO annotation using genes whose differentially expressed were significant in recMFAP5 versus DMSO group (red: appearing peak DGEs, blue: disappearing peak DGEs). (C) GSEA analysis revealed genes of Notch1 pathway in iCCA cells treated with recMFAP5. (D) GSEA analysis revealed genes in the canonical WNT signaling pathway in iCCA cells treated with recMFAP5. (E) Colony formation and CCK-8 assay showed the colony forming ability and proliferation ability of iCCA cells treated with DMSO/recMFAP5/FLI-06/Crenigacestat. (F) Western blot results showed the expression level of Notch1 pathway targeted genes in iCCA cells treated with DMSO/recMFAP5/FLI-06/Crenigacestat. (G) The gene peaks results revealed changes in different groups (blue: DMSO group, red: recMFAP5 group). Data were presented as mean ± SD and compared by t-test. ***p*＜.01, ****p*<.001


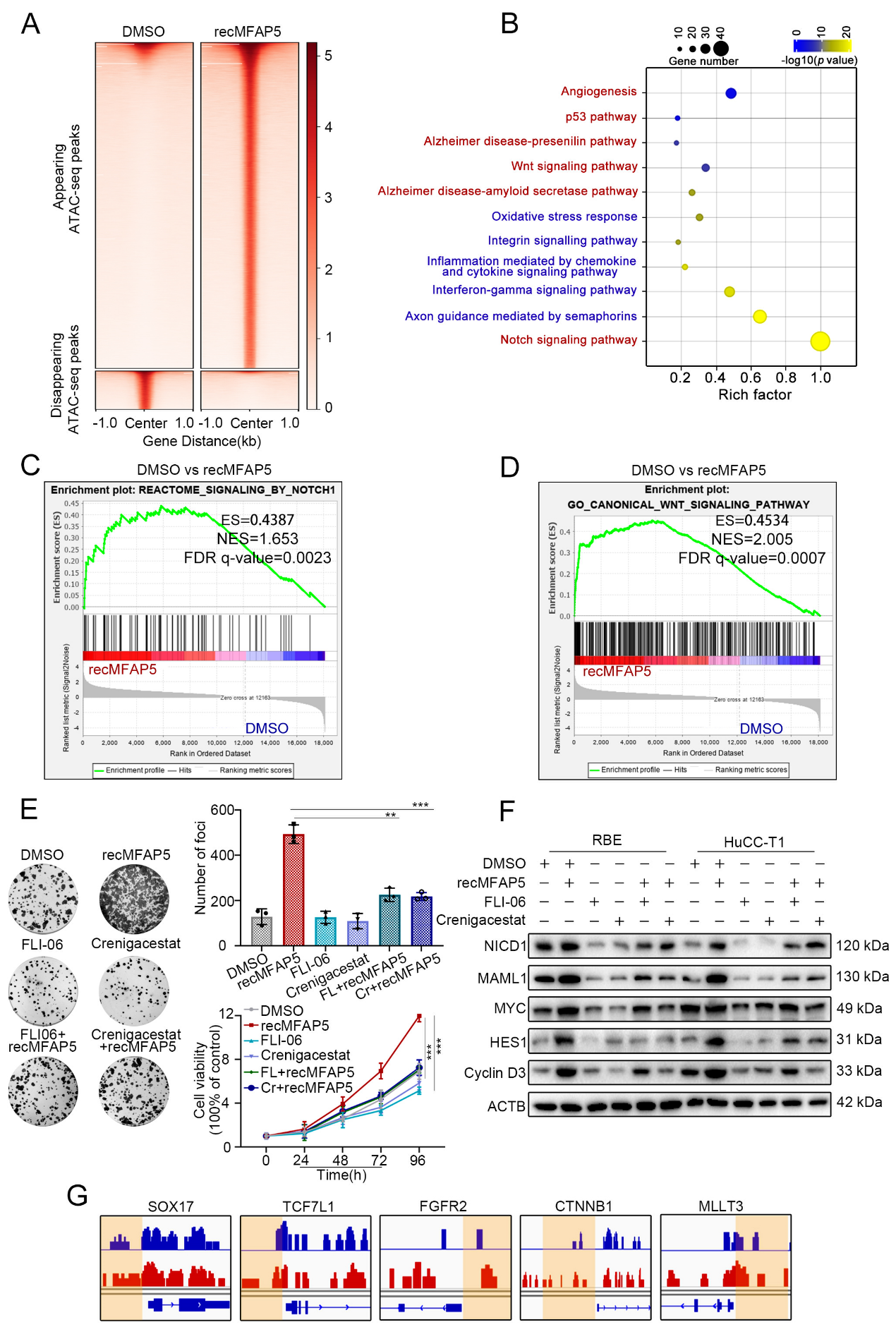


**Supplementary Figure 12. Outcome of NicheNet’s ligand activity prediction**

(A)tSNE plots for the cell type identification of 4946 high-quality single cells obtained from iCCA tumor tissue after singleR labeling. (B) tSNE plots for the cell type identification of 14447 high-quality single cells obtained from iCCA tumor-adjacent tissue after singleR labeling. (C) Results were shown for the 18 (of 125) CAF-ligands best predicting the NOTCH1/WNT pathway downstream gene list. As the ligand activity ranking metric, we used the Pearson correlation coefficient between prior regulatory potential scores and downstream gene list assignments (n= 14 genes, of which 7 belong to the NOTCH1 pathway and 7 belong to WNT pathway). This Pearson correlation indicates the ability of each ligand to predict the pathway target genes, and better predictive ligands are thus ranked higher. (D) NicheNet’s ligand–target matrix denoting the regulatory potential between CAF-ligands and target genes from the NOTCH1 (n=6, included CCND1, HES1, MYC, RBPJ, NOTCH1, CCN3) and WNT (n=2, included CTNNB1, SOX17) pathway. (E) Results were shown for the 20 (of 121) fibroblast-ligands best predicting the NOTCH1/WNT pathway downstream gene list. (F) NicheNet’s ligand–target matrix denoting the regulatory potential between fibroblast-ligands and target genes from NOTCH1 and WNT pathway.

**
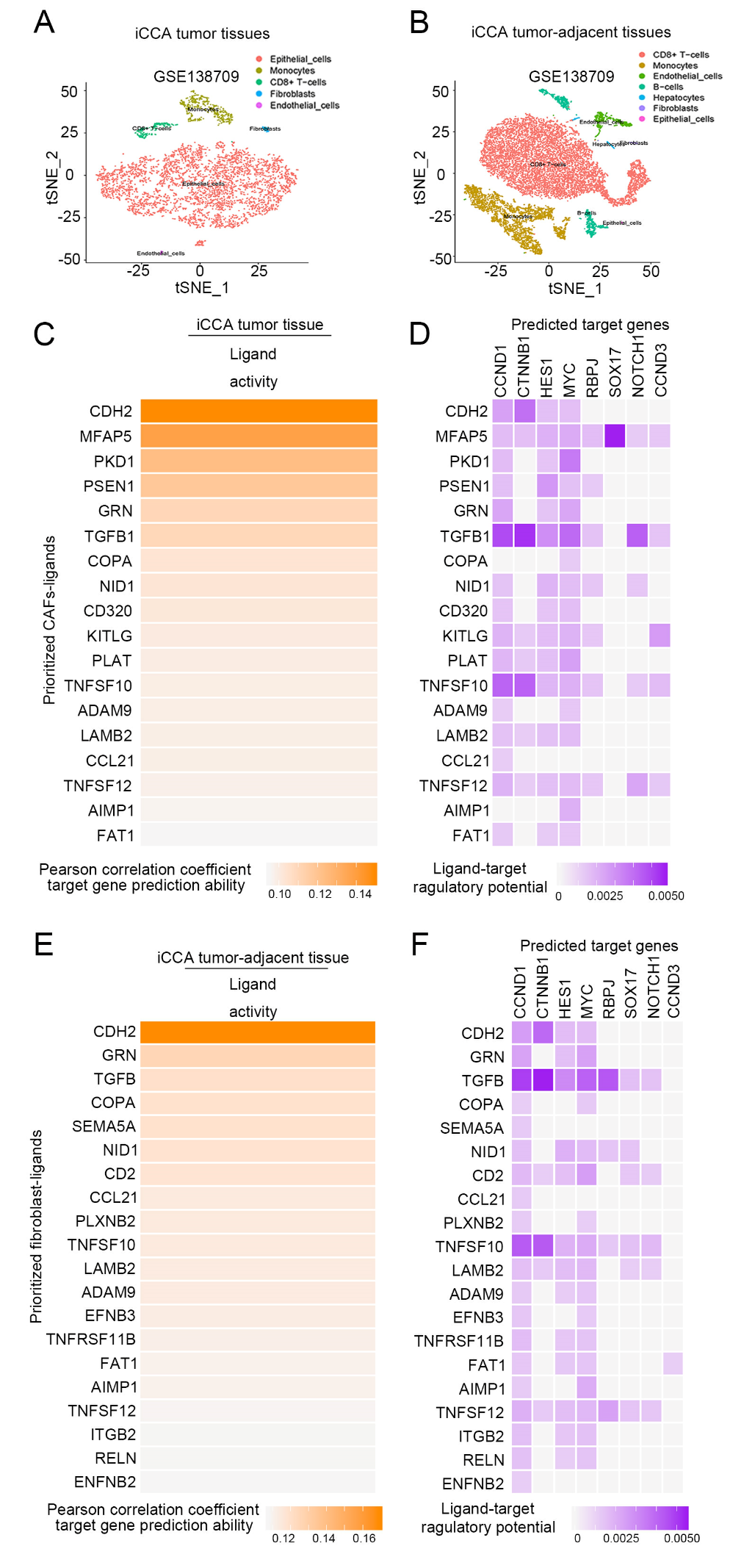
**

**Supplementary Figure 13. The quantification of western blot for Figure 1 and Figure 5**

(A) Gray value analysis showed the relative AGTR1 expression to GAPDH in CAFs#1-#6, RBE, HuCC-T1 and NF-1 (related to Figure 1F). (B) The relative AGTR1 expression level to ACTB of ^unsorted/AGTR1+/AGTR1-^CAFs#1, #2 was showed in the left histogram. The relative MFAP5 expression level to ACTB of ^unsorted/AGTR1+/AGTR1-^CAFs#1, #2 was showed in the right histogram (related to Figure 5A). (C, D, E) The relative MFAP5 expression level to ACTB of ^AGTR1+^CAFs#1, #2 after treated with losartan (300μM)/valsartan (100μM), Ang II (10nM) or XMU-MP-1 (50/100nM) was showed in gray value histogram (related to Figure 5B, 5C, 5D). (F, G) The relative LATS1, p-LATS1, YAP1, p-YAP1 and MFAP5 expression level to ACTB of ^AGTR1+^CAFs#1, #2 after treated with losartan (300μM)/valsartan (100μM) or XMU-MP1 (100nM) was showed in gray value histogram (related to Figure 5E, 5F). (H, I) The relative YAP expression in cytosol or nucleus to GAPDH or H3 of ^AGTR1+^CAFs#1, #2 after treated with XMU-MP1 (100nM) was showed in gray value histogram (related to Figure 5G, 5H). Data were presented as mean ± SD (n=3) and compared by t-test. **p*<.05, ***p*<.01, ****p*<.001,*****p*<.0001, versus DMSO group.

**
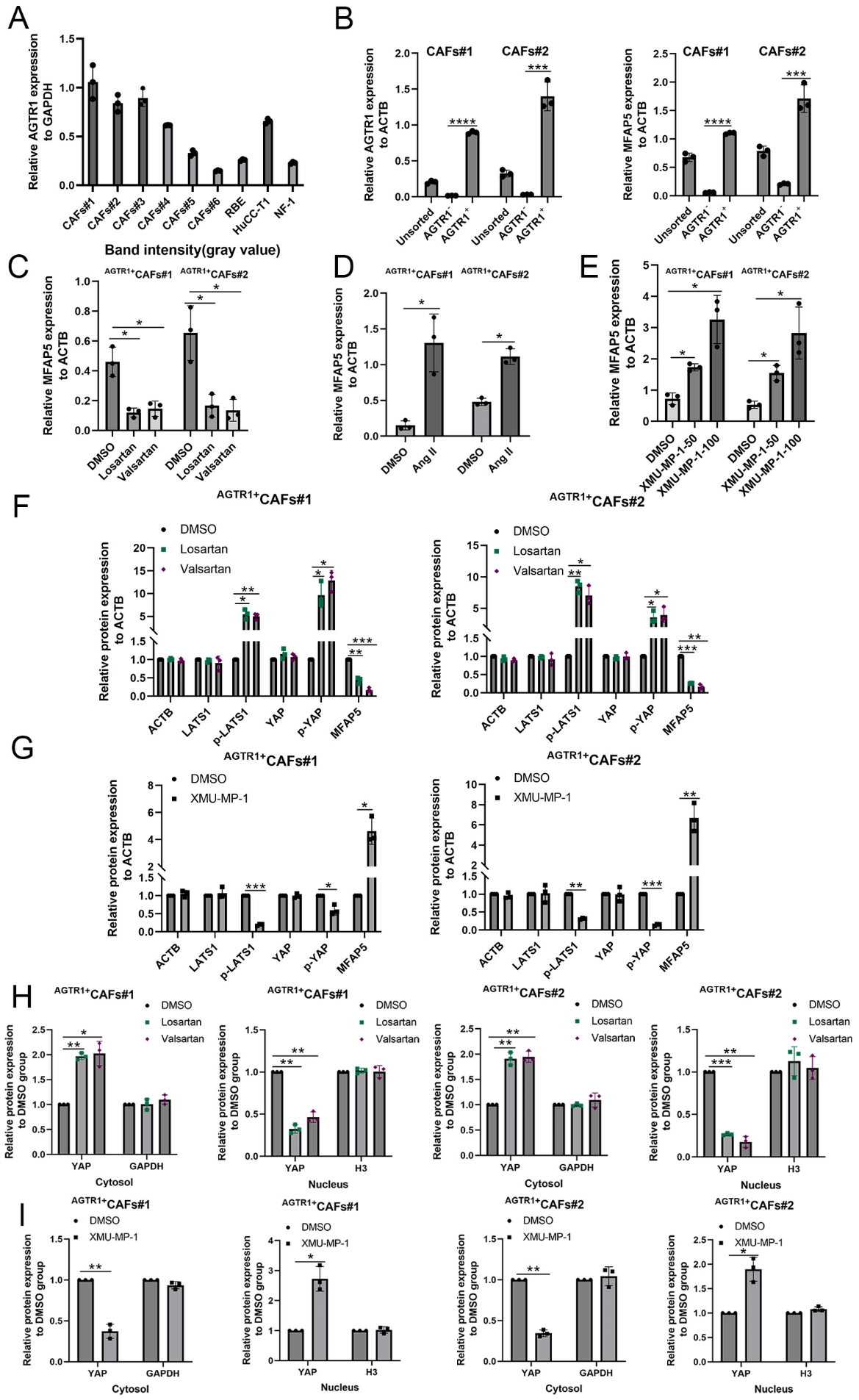
**

**Supplementary Figure 14. The AGTR1 protein level of 5^th^-10^th^ passages of CAFs#1-6**

(A，B， C，D，E，F) The AGTR1 protein level of 5^th^-10^th^ passages of CAFs#1-6

was detected by western blot. The relative AGTR1 expression level to GAPDH of 5^th-^10^th^ passages of CAFs#1-6 was shown in the matching histograms (under western blot assay).

**
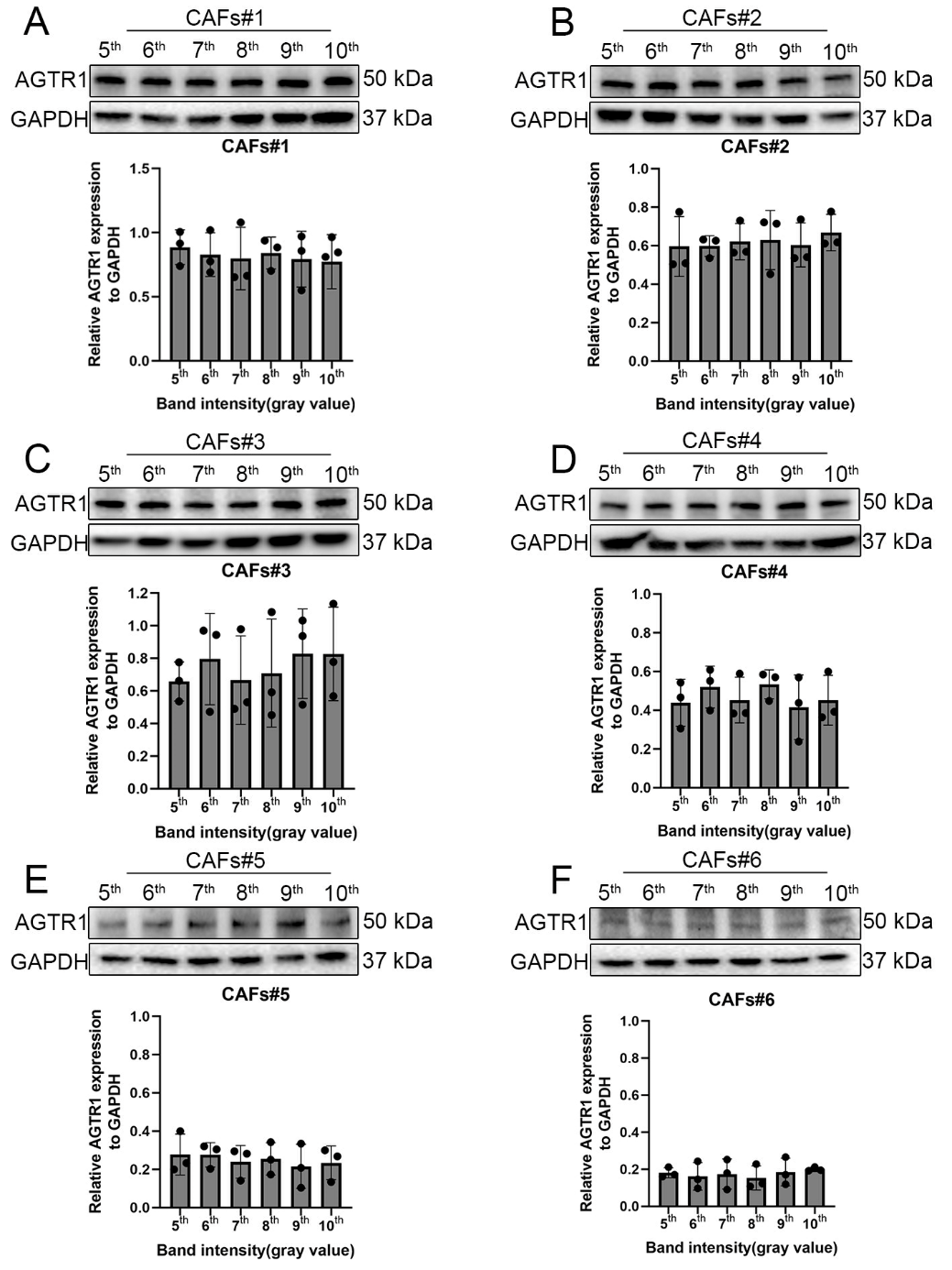
**

**Supplementary Figure 15.** **MFAP5, an ECM glycoprotein, was associated with the prognosis of iCCA and could be identified as a diagnostic indice and therapeutic target of iCCA**

(A, B) Prognostic results based on MFAP5 expression level in iCCA tissues (the median of IHC score=4, n=208. (C) MFAP5 serum level (ELISA) in healthy volunteers, iCCA patients and hepatocellular carcinoma (HCC) patients (n=8,32,13 respectively). (D) Area under the curve (AUC) analysis of iCCA patients, healthy volunteers and HCC patients based on MFAP5 serum level. (E) MFAP5 serum level (ELISA) of pre-operation and 7 days after operation in iCCA patients (n=64). Data was presented as mean ± SD and compared by t-test. **p*＜.05, ***p*＜.01, ****p*＜.001.


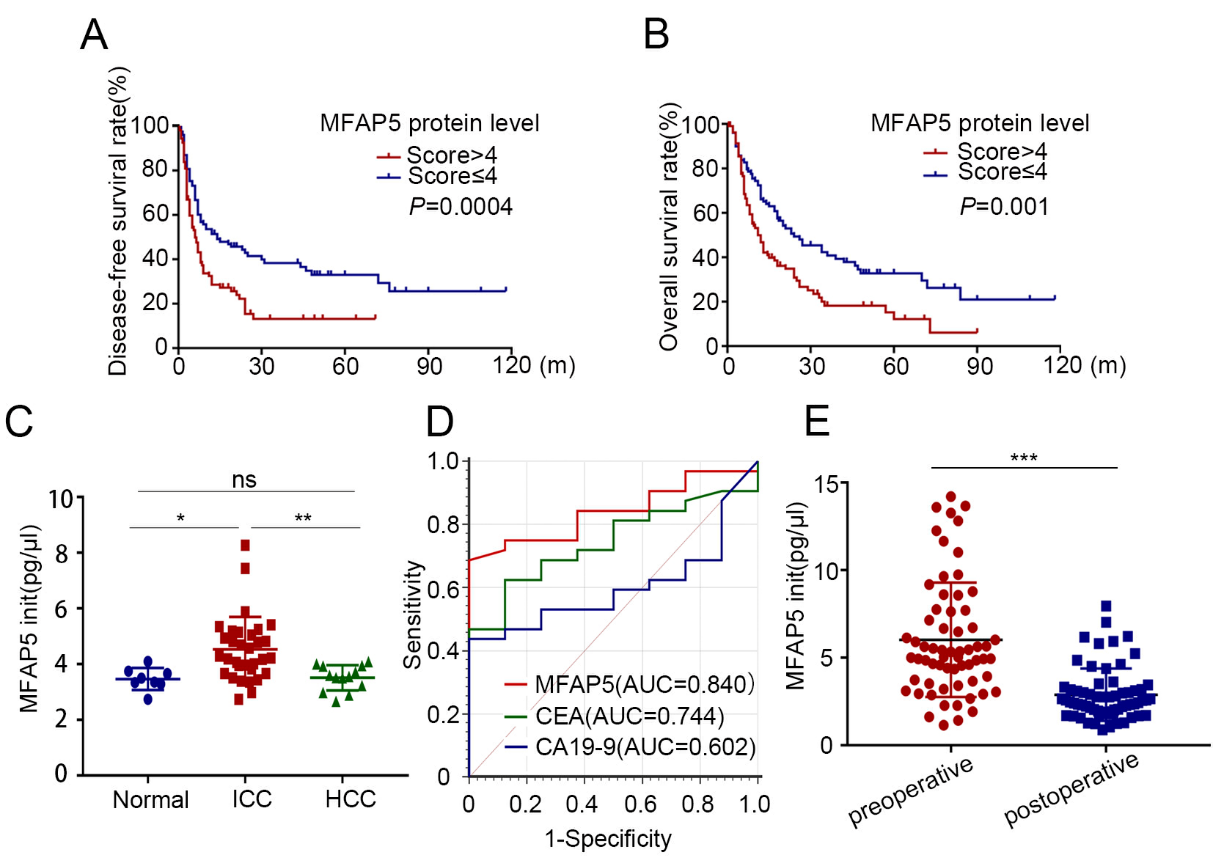


**Supplementary Table 1. The clinic pathological characteristics of 91 iCCA patients**

| Characteristics | Values |
| --- | --- |
| Age, year (mean ± SD) | 63.5 ±9.63 (37-81) |
| Gender (male/female) | 55/36 |
| Receiving antihypertensive drugs (Yes/No) | 71/20 |
| Tumor length, cm (≤5/＞5) | 51/40 |
| 8^th^ AJCC TNM stage (Ia/Ib/II/IIIa)  Tumor grade (unascertained/1/2/3) | 34/18/19/20  18/15/51/7 |
| Tumor recurrence, months (mean ± SD) | 21.76 ±18.60 (1-76) |
| Overall survival, months (mean ± SD) | 27.91 ± 18.10 (5-76) |

**Supplementary Table 2. Number of patients receiving antihypertensive drugs**

| Characteristics | Numbers |
| --- | --- |
| ACEIs^a^ |  |
| Captopril | 2 |
| Perindopril | 2 |
| ARBs^b^ |  |
| Losartan | 6 |
| Valsartan | 10 |
| Telmisartan | 6 |
| Irbesartan | 11 |
| CCB^c^ |  |
| Amlodipine | 14 |
| Nifedipine | 22 |
| Felodipine | 1 |
| ^a^ACEIs+CCB | 3 |
| ^b^ARBs+CCB | 15 |
| ^b^ARBs+CCB+β-blockers | 7 |
| ^b^ARBs+ Diuretics | 3 |
| ^c^CCB+β-blockers | 4 |

Abbreviations: ACEIs: angiotensin-converting enzyme inhibitors; ARBs: angiotensin II receptor blockers; CCB: calcium-channel blockers.

**Supplementary Table 3. Sequences of primers and shRNAs used in this study**

| Names | Sequences |
| --- | --- |
| MFAP5-shRNA-1# | GGACCCAAGGTGCTGCTGTTT |
| MFAP5-shRNA-2# | CGTATGTACATCGTCAACAAG |
| Control-shTRC | GCTTCGCGCCGTAGTCTTA |
| GAPDH-primer-F | GAGTCCACTGGCGTCTTCAC |
| GAPDH-primer-R | ATCTTGAGGCTGTTGTCATACTTCT |
| MFAP5-primer-F | GGGTCAATAGTCAACGAGGAGA |
| MFAP5-primer-R | CTGTAGCGGGATCATTCACCA |
| AGTR1 -primer-F | GGCTATTGTTCACCCAATGAAGT |
| AGTR1 -primer-R | TGGGACTCATAATGGAAAGCAC |
| CCN2-primer-F | TGGGACTCATAATGGAAAGCAC |
| CCN2-primer-R | CTTGTGGCAAGTGAATTTCC |
| CCN1-primer-F | AAGAAACCCGGATTTGTGAG |
| CCN1-primer-R | GCTGCATTTCTTGCCCTTT |
| ANKRD1-primer-F | GCCTACGTTTCTGAAGGCTG |
| ANKRD1-primer-R | GTGGATTCAAGCATATCACGGAA |

**Supplementary Methods**

1. **Immunohistochemical (IHC) staining**

Slides containing the sections were stained with anti-AGTR1 (1:200, #25343-1-AP, proteintech), and anti-MFAP5 (1:800, #15727-1-AP, proteintech) antibodies. Staining intensity (negative, 0; mild, 1; moderate, 2; severe, 3) and proportion of positive cells (negative,0; ≤10%, 1; >10 and ≤33%, 2; >33 and ≤66%, 3; >66%, 4) were quantified respectively. Two experienced pathologists scored the stained tissues independently.

1. **Immunofluorescence staining assay for CAFs**

Cells were seeded on glass coverslips and then fixed with 4% paraformaldehyde for 20 minutes and washed three times with PBS for 5 minutes each. Cells were permeabilized with 0.1% Triton X‐100 (Solarbio #T8200) for 10 minutes and washed three times with PBS for 5 minutes each. They were then blocked in 10% goat serum (Thermo Fisher Scientific #16210072) for 1 hour at room temperature. Incubation with α-SMA antibody (1:500, Abcam ab32575) was carried out at 4°C for 16 hours. Nuclei were counterstained with DAPI (Thermo Fisher Scientific #P36931). Fluorescent images were obtained using a ZEISS LSM710 laser scanning confocal microscope and ZEN 2.3 software.

1. **Extraction and processing of gene expression omnibus (GEO)**

Quantified count matrices from GSE142784 (GSM4240155, GSM42401560) and GSE138709 (GSM4116580 and GSM4116583) were merged in R and analyzed with the Seurat package (v 4.2.0) [1]. First, cells with <200 measured genes, or >7000 measured genes, or >10% mitochondrial counts were removed from the dataset. After filtering, data in each cell was normalized (function NormalizeData, method = ‘LogNormalize’, scale.factor = 10,000), the 2,000 most variable genes were obtained (function FindVariableFeatures, selection.method = “vst”, nfeatures = 2000). We then identify anchors using the FindIntegrationAnchors function with these 2,000 most variable genes. Subsequently, these anchors were used to integrate the two datasets together with IntegrateData function. 20 principal components were used for graph-based clustering (resolution = 0.1) and TSNE dimensionality reduction was computed[2]. All steps were performed using functions implemented in the Seurat package with default parameters, except where mentioned. Markers for each cluster were identified using the FindAllMarkers function with parameters of ‘test.use = “wilcox”, min.pct = 0.1 ’. Cell annotation was implemented by using singleR, a reference-based automatic annotation method [3].The clusterProfiler package (v 4.2.2) was employed to conduct GO enrichment analysis. Cell-cell interaction (CCI) between preselected cell types were predicted based on existing knowledge on ligand-to-target signaling paths by NicheNet method [4]. Using preselected target genes, we assessed how well ligands expressed by the sender cell could predict changes in gene expression in the receiver cell and prioritized ligands based on their effects with function predict ligand activities. Then, we inferred active target genes and receptors of the ranked ligands. All results were visualized with make heatmap ggplot.

1. **Assay for Transposase Accessible Chromatin with high-throughput sequencing**

Chromatin preparation Nuclei was prepared from 4 × 10^4^ cells. Library amplification was performed using the NEBnext High Fidelity 2× PCR Master Mix (#M0541S, New England Biolabs) according to previously published PCR conditions. ATAC-seq library preparations were sequenced using single-end 50-bp reads on the Illumina HiSeq 2000 platform. Raw reads were adaptor-trimmed using Trim Galore (v0.2.5) and aligned to the genome with Bowtie (v1.0.1) with the m1 option enabled to allow only uniquely aligned high-quality reads. Peaks were called using the MACS2 software (v2.1.0.20140616) with the options −q 0.05 to retain significant peaks and shift size 50 to account for the transposase fingerprint, while default parameters were used for other options.

1. **Protocol for calculating stromal proportions**

The stroma proportion of iCCA tissues was calculated by the ImageJ Software. First, open the Masson trichrome staining image by ImageJ (File→Open→Select picture). Then follow these steps: Image→Adjust→Color Threshold. Adjust the “Hue” to select the “stroma” and “tumor cells” respectively as following pictures shown. Then click “Analyse→Measure” to calculate the selected area (Area_stroma_ and Area_tumor_). The proportion of stroma was calculated by the following formula:

Proportion_stroma_=Area_stroma_/ (Area_stroma_+Area_tumor)_

**REFERENCE**

1. Satija, R.; Farrell, J.A.; Gennert, D.; Schier, A.F.; Regev, A. Spatial reconstruction of single-cell gene expression data. Nat Biotechnol 2015, 33, 495-502, doi:10.1038/nbt.3192.

2. Tirosh, I.; Izar, B.; Prakadan, S.M.; Wadsworth, M.H., 2nd; Treacy, D.; Trombetta, J.J.; Rotem, A.; Rodman, C.; Lian, C.; Murphy, G.; et al. Dissecting the multicellular ecosystem of metastatic melanoma by single-cell RNA-seq. Science 2016, 352, 189-196, doi:10.1126/science.aad0501.

3. Aran, D.; Looney, A.P.; Liu, L.; Wu, E.; Fong, V.; Hsu, A.; Chak, S.; Naikawadi, R.P.; Wolters, P.J.; Abate, A.R.; et al. Reference-based analysis of lung single-cell sequencing reveals a transitional profibrotic macrophage. Nat Immunol 2019, 20, 163-172, doi:10.1038/s41590-018-0276-y.

4. Browaeys, R.; Saelens, W.; Saeys, Y. NicheNet: modeling intercellular communication by linking ligands to target genes. Nat Methods 2020, 17, 159-162, doi:10.1038/s41592-019-0667-5.
